# Supplementary material for: Subicular spatial codes arise from predictive mapping
Source: Nat Commun. 2026 Aug 31;17:10373. doi: 10.1038/s41467-026-76686-y (PMC13627671; doi:10.1038/s41467-026-76686-y)
Supplement: Supplementary file 1 [file 41467_2026_76686_MOESM1_ESM.pdf]

Supplementary information

Additional RSA results

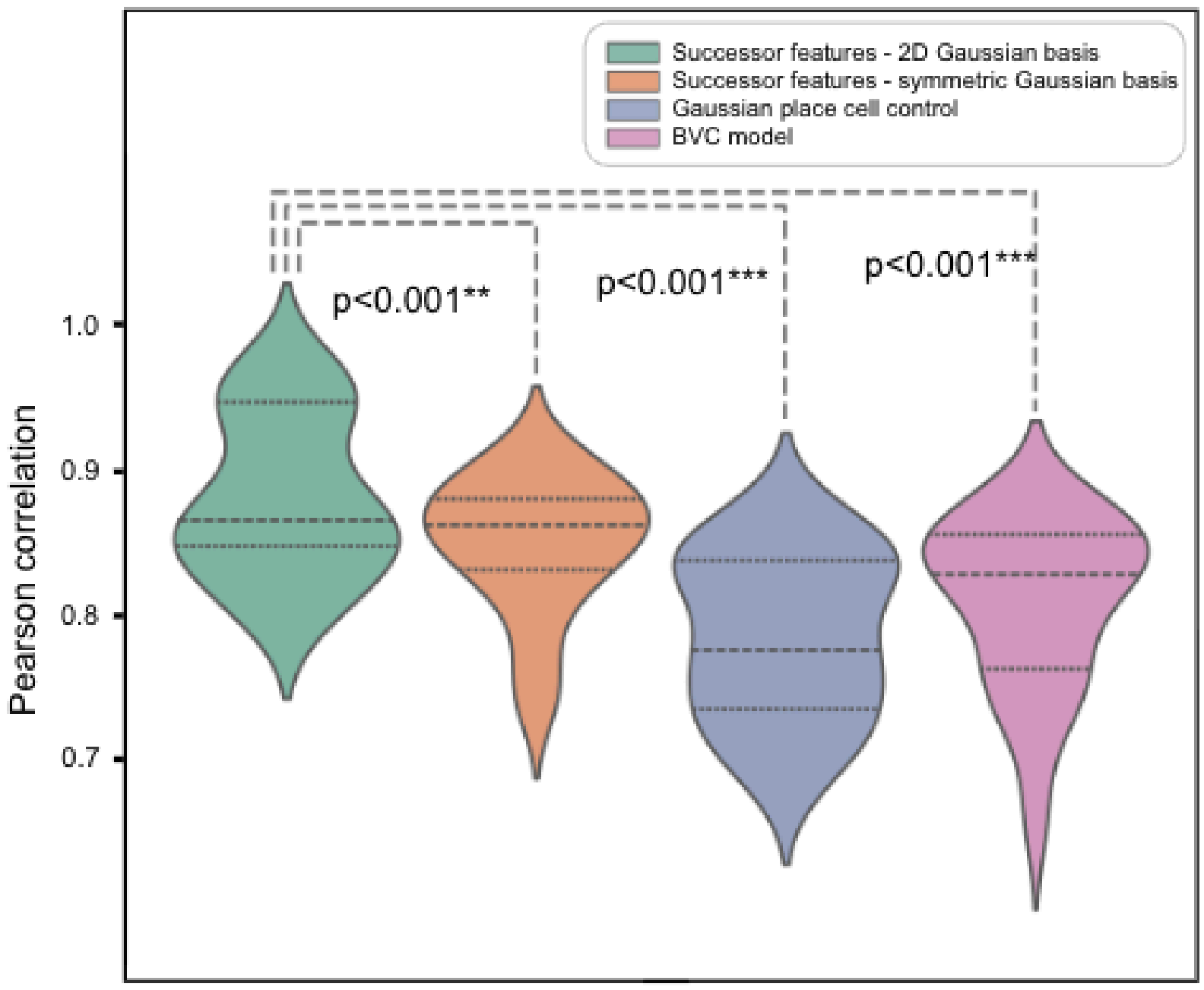

Supplementary Fig. 1 Additional RSA correlations to subiculum recordings. All subicular recordings are better fit by successor features built on biologically-scaled basis features and real rodent trajectories, than successor features built on uniform and symmetric Gaussian place cell bases and real rodent trajectories. However, all subicular recordings are distinctly better fit by both populations of successor features than either Gaussian basis features, or traditional 'boundary vector cells'[1]. *p<0.05; **p<0.01;***p<0.001.

Remapping simulation across different discount factors

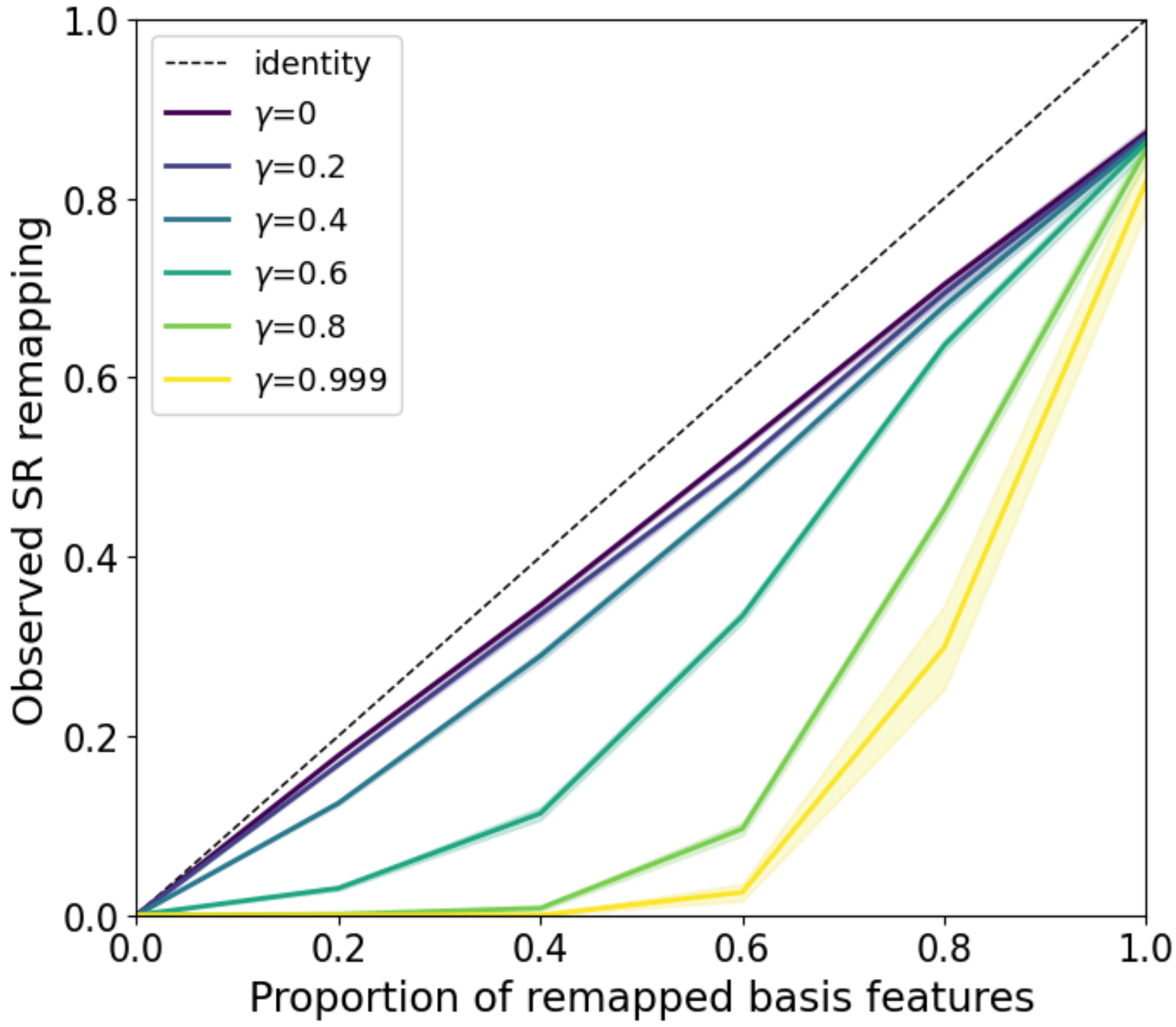

Supplementary Fig. 2. Resistance to remapping scales with predictive temporal horizon. Observed successor feature remapping as a function of the proportion of remapped basis features, across a range of temporal horizons (γ = 0 to 0.999). At short horizons, successor feature remapping closely tracks basis feature remapping (dashed identity line). As the temporal horizon increases, successor features become progressively more resistant to upstream perturbations, with long-horizon representations (γ = 0.999) substantially buffered against partial remapping of the input. solid lines and shaded regions indicate mean and standard deviation calculated across 10 random seeds.

Successor features skew

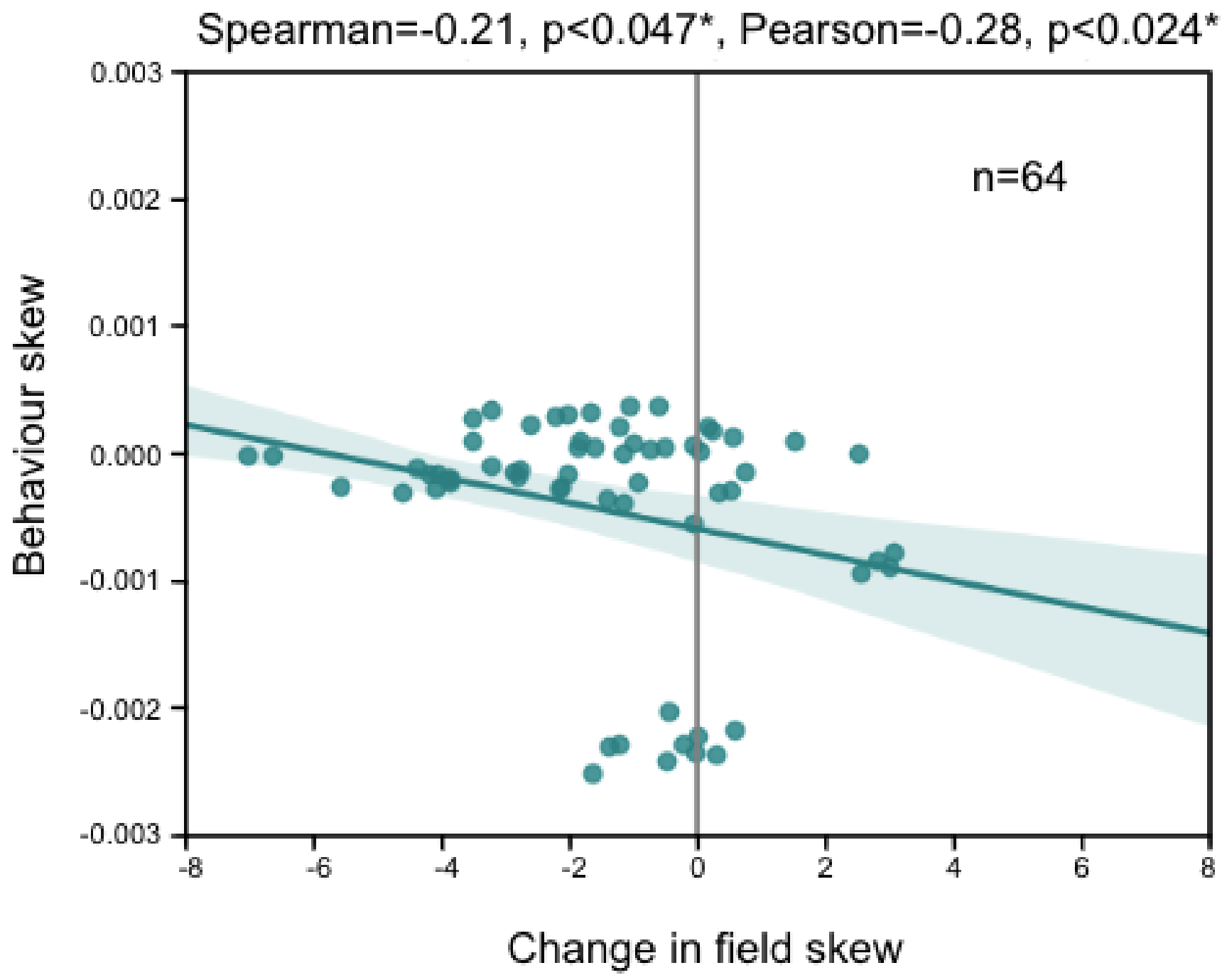

Supplementary Fig. 3 Successor features skew against the dominant axis of an animal's trajectory in a 2D environment. As previously reported in 1D environments, boundary-tuned successor features that are formed from the two longest distance consecutive rodent trajectories skew negatively against the animal's dominant axis of travel parallel to the field-responsive wall. Shaded regions indicate 95% confidence intervals. *p<0.05; **p<0.01;***p<0.001.

Basis features underpinning types of successor features

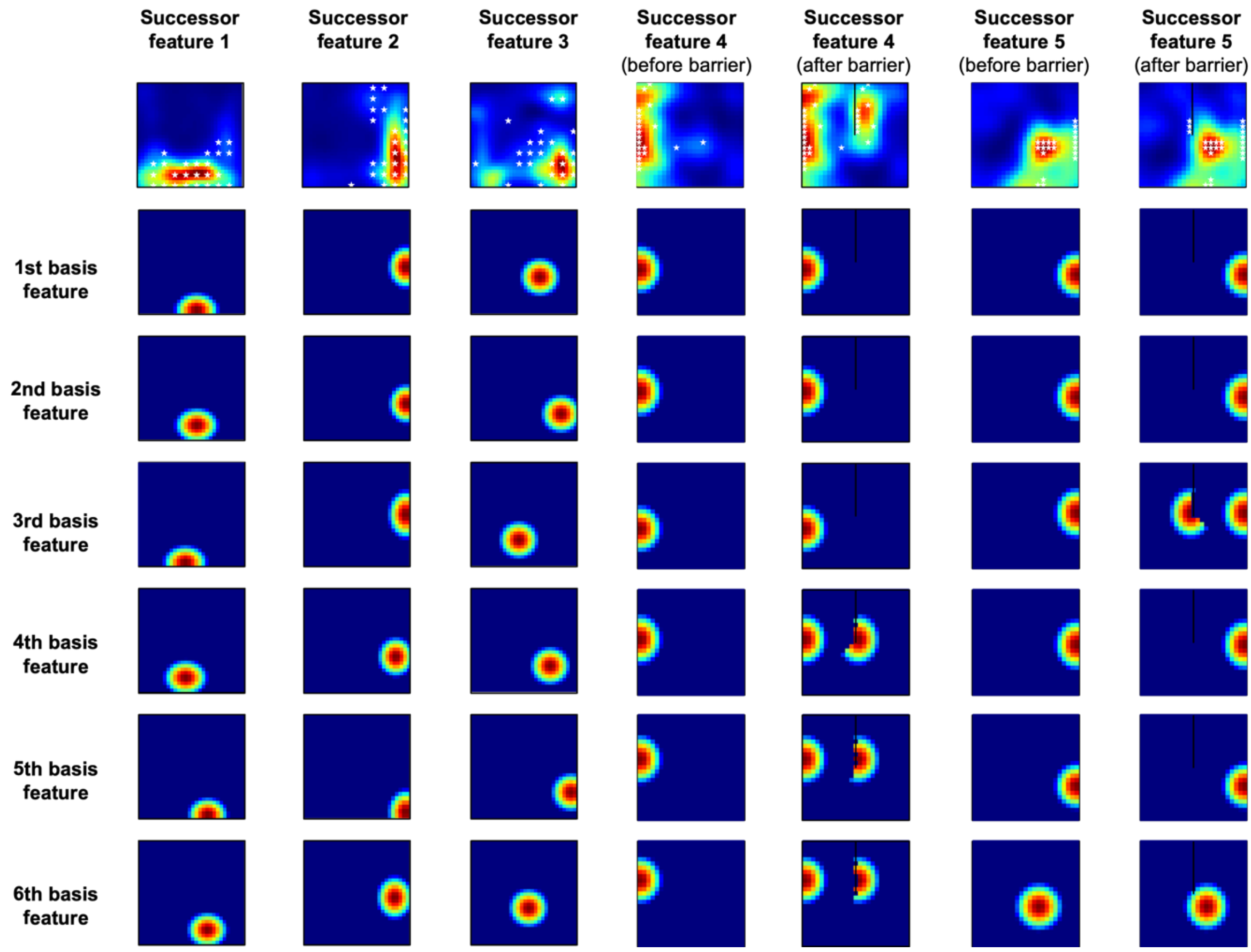

Supplementary Fig. 4 Spatial distribution of top contributing basis features to a range of successor features. Example spatial tunings of successor features (top row, with white stars denoting field centres for largest contributing basis features) and their largest contributing basis features (columns – ordered by weight). Successor features 1 and 2 are classified as boundary responsive cells with tunings to the south and east wall respectively, with top contributing basis features primarily spanning these regions. Successor feature 3 is classified as a corner cell, as a result of top contributing basis features clustering in corner locations. Successor feature 4 is classified as a boundary responsive cell tuned to the west wall, that duplicates upon barrier insertion due to a high proportion of its top contributing basis features themselves duplicating. In contrast, successor feature 5 does not show this duplication and has top contributing basis features that largely do not duplicate.

Successor features built on CA1 recordings

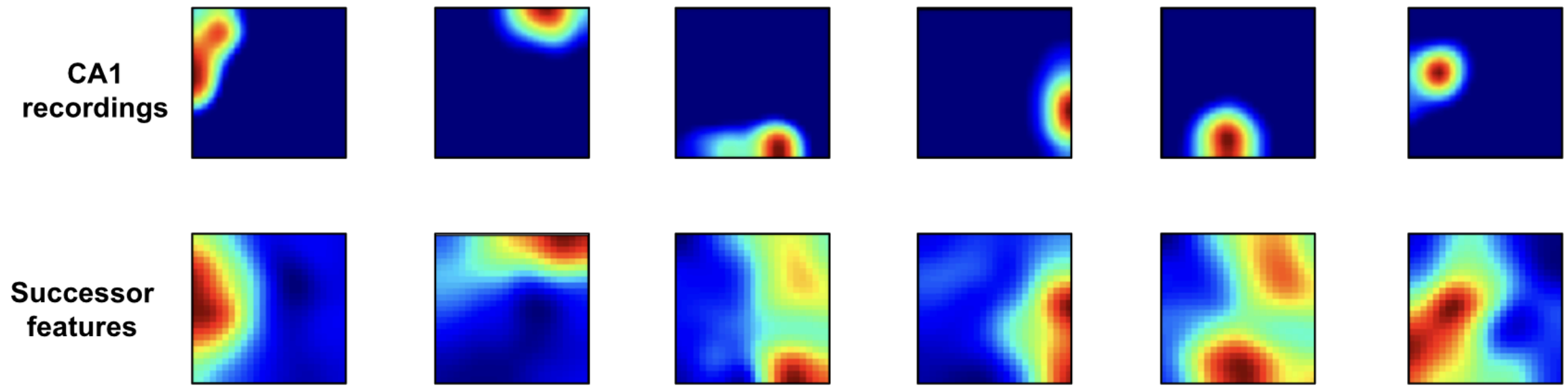

Supplementary Fig. 5 Single population of successor features built on real CA1 recordings. For one environment (Muessig and colleagues[21,60,61]), 67 CA1 place cells were recorded over two consecutive trials, and we use these along with their longest distance rodent-trial trajectory to build a single population of 67 successor features which reproduce the topology of successor features built on biologically plausible basis features. 4.5% of these resulting successor features are classified as corner cells, and 74% are classified as boundary vector cells.

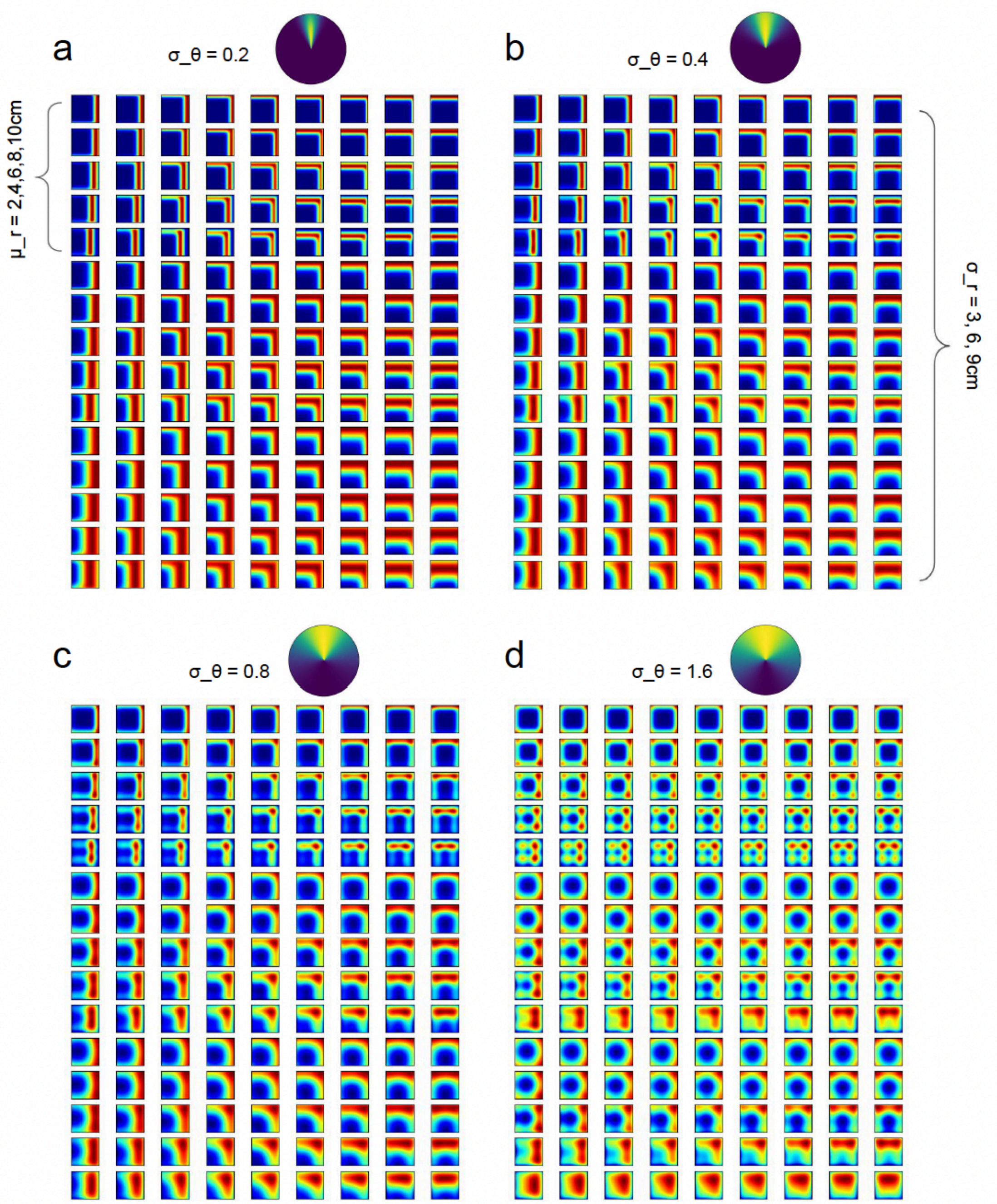

Supplementary Fig. 6 The boundary vector cell model produces fields that resemble multi-lobed corner responses for extreme angular tuning parameters. Boundary vector cell populations and their circular receptive fields covering nine preferred angular directions, five preferred distance tunings (μ_r) and three radial tuning widths (σ_r), shown at four angular tuning widths (σ_θ) of (**a**) 0.2, (**b**) 0.4, (**c**) 0.8 and (**d**) 1.6 radians — i.e. the canonical value used by Hartley et al.[18] (0.2 radians) and 2×, 4× and 8× that value. Fields that resemble multi-lobed corner responses only begin to emerge at σ_θ = 1.6 radians, well outside the parameter range of the original model; however, none of these units passed the shuffling criterion applied by Sun et al.[9] and none were therefore classified as corner cells. Note that at this tuning the model essentially predicts responses against all barriers regardless of their orientation relative to the agent.

Appendix

Derivation of Successor feature learning rule

The learning rule (Equations 1-2) is derived using semi-gradient temporal difference learning[2]. Starting with the definition that successor features $\boldsymbol{\psi}(s)$ represent the expected discounted future occupancy of basis features $\boldsymbol{\varphi}(s)$:

$\boldsymbol{\psi}(s) = E[\Sigma\ \gamma^t \boldsymbol{\varphi}(s_t)\ |\ s_0=s]$

Thus,

$\boldsymbol{\psi}(s_t) = \boldsymbol{\varphi}(s_t) + \boldsymbol{\psi}(s_{t+1})$

We seek a matrix M such that $\boldsymbol{\psi}(s) = M\boldsymbol{\varphi}(s)$. Using semi-gradient descent, we minimise square of the temporal difference error: $\boldsymbol{\delta}^2 = [\boldsymbol{\varphi}(s_t) + \gamma\boldsymbol{\psi}(s_{t+1}) - \boldsymbol{\psi}(s_t)]^2$

The key insight is that we only take the gradient with respect to $\psi(s_t)$, treating $\psi(s_{t+1})$ as fixed (hence "semi-gradient"). This yields: $\partial\boldsymbol{\delta}^2/\partial M = -2\boldsymbol{\delta}\boldsymbol{\varphi}(s_t)^T$

Following the negative gradient gives our update rule: $M \leftarrow M + \alpha[\varphi(s_t) + \gamma\psi(s_{t+1}) - \psi(s_t)]\varphi(s_t)^T$

References

1. Hartley, T., Burgess, N., Lever, C., Cacucci, F. & Keefe, J. O. Modeling Place Fields in Terms of the Cortical Inputs to the Hippocampus. *Hippocampus* **379**, 369–379 (2000).
2. Sutton, R. S. & Barto, A. G. *Reinforcement Learning: An Introduction*. (MIT Press, 2018).
