# Supplementary material for: Subicular spatial codes arise from predictive mapping
Source: Nat Commun. 2026 Aug 31;17:10373. doi: 10.1038/s41467-026-76686-y (PMC13627671; doi:10.1038/s41467-026-76686-y)
Supplement: Supplementary file 2 [file 41467_2026_76686_MOESM2_ESM.pdf]

nature portfolio

Peer Review File

Subicular spatial codes arise from predictive mapping

Corresponding Author: Mx Lauren Bennett

This file contains all reviewer reports in order by version, followed by all author rebuttals in order by version.

Version 0:

Reviewer comments:

Reviewer #1

(Remarks to the Author)
In this paper Bennet et al. present a successor representation (SR) model to propose that subicular spatial codes might arise from predictive mapping. The SR is learned over a basis of CA1-like place-cells and shaped by rodent behavioral biases (e.g., thigmotaxis and corner occupancy). Using a long predictive horizon ($\gamma \approx 0.995$; ~16–20 s), their SR model reproduces hallmark subicular responses such as boundary and corner cells, near-wall distance bias, directional clustering orthogonal to walls, resistance to moderate remapping, and barrier-induced field duplication. The SR model matches population statistics better than a boundary-vector-cell model or a Gaussian place-cell control. Based on these findings the authors hypothesize a temporal hierarchy in which CA1 encodes short-horizon predictions while subiculum integrates over longer timescales.

Fig. 2f reports only the differences between models and subiculum, without giving the actual proportion of boundary cells in the subicular data. Please report these the empirical values.
Each modeled subicular unit, SFi, is trained to generate a policy-weighted, temporally discounted expectation of future activity of a corresponding basis place cell, φi. Therefore, SFi maps where the agent is most likely to enter φi's firing field in an exponentially discounted time window (set by γ) under the empirical training policy. To clarify how much of the SF boundary code is predicted vs. inherited from the CA1-like basis, please add a visualization of the spatial distribution of the basis (place-cell) fields that served as cumulants for the subset of SF units that were classified as boundary cells and corner cells. Ideally, this visualization should be done separately for each directional mode of the boundary cell subpopulation and for each corner preference of the corner cell subpopulation. Similarly, in the barrier-duplication analyses, please provide visualizations of the spatial distribution of the basis (cumulant) place-cell fields for duplicating vs. non-duplicating SF (subicular) units.

Fig. 4 shows that subicular fields shift their center-of-mass against the dominant axis of travel across consecutive trials. This is argued to be consistent with the SR account, but there is no companion simulation. If these data are to be cited as support for the model, then an SR simulation that reproduces the effect should be provided.

Figure 7 shows that small γ yields SFs resembling the original basis. While this is useful as a sanity check, it seems somewhat tautological that small γ will transform the basis set less than large γ. A more probative test of the paper's interesting claims about hierarchical processing would be to simulate a two-stage cascade (φ -> small γ SF φ -> large γ SF) and compare the final layer SFs to the current one-stage setup (φ -> large γ SF).

The results are interpreted as showing that subiculum encodes a longer predictive horizon than CA1 (~20 s vs. ~1–2 s). However, the open-field foraging paradigm incorportaes a stable policy (thigmotaxis, corner occupancy) with constant state values; there is no need to revalue specific states for decision-making. SRs were originally proposed to enable flexible revaluation in dynamic environments with changing reward contingencies. Thus, the apparent fit of subiculum to long predictive horizons may partly reflect the behavioral context (stable policy and state values stable) rather than general coding principles. A stronger test would use an experimental/simulation paradigm in which the SR can perform its intended function of rapid revaluation with slower, policy-dependent SR updating. For example, it might be shown that splitter-like SFs can emerge in maze tasks under reversal learning. Please consider in simulations or discussion how task demands (decision timescale, contingency changes) might shift the effective predictive horizon.

(Remarks on code availability)

Reviewer #2

(Remarks to the Author)
The successor representation (SR) is a dominant model for the function of place cells in the hippocampus, and has been demonstrated to explain the the properties of place cells in CA1. The current paper demonstrates that in many aspects, subiculum recordings could also be modeled by the SR, and that many predictions could be explained by such a model, and that the main difference between CA1 and subiculum is the time constant of prediction, related to the predictive horizon of the representation. I am very fond of the SR and am happy to see it applied to the subiculum, however I have several reservations to the current paper, which need to be checked, in order to ascertain that indeed it is appropriate to connect the SR model to the subiuclum:

1) One major difference between CA1 and subiuclum is in the pattern of remapping. How do the authors explain the difference using SR - that subiuclum in principle does not remap, while CA1. Is there any way to link the SR framework to understanding this question?

2) Another textual issue (e.g. line 62) - to claim that SR is a class of a predictive algorithm is not completely precise, as it is only a predictor of action, while not being a predictor, for example, of the sensory input. I would make this distinction in the text.

3) Fig 1a - how does the choice of the basis feature affect the results? Instead of taking synthetic basis features, why not use rate maps of real place cells?

4) Figure 2 - Using the procedure of Muessig et al. is compelling, however I do not believe this is optimal in this case, because if the question is to compare BVC models to SR models, then these are the models that should be compared, no? What is done in the paper is to show the distribution of BVC parameters in three different models, which does not answer the question whether BVC models are better or worse than models based on SR. Thus - what should be done is to create a whole set of SR models (with different discounts into the future) and a whole set of different BVC models (with different radii and a different opening angle) and then to test these on the data, preferably using a training set (to find the optimal model for each category) and then testing using a test set.
A corollary of that is the strange over-estimation in line 163 - a more "kosher" training set and test set would have probably avoided such issues.

5) line 179 - "Conversely, the place cell control model was only matched to perpendicular vectorial tunings" - isn't that an artifact of the way the model was constructed? Please clarify.

6) Figure 3 - how is the resistance to remapping consistent with the ideas about SR in CA1? Please clarify.

7) Line 244 - the phenomenon of skew mentioned here was also shown for grid cells by Keinath, A. T., Epstein, R. A., & Balasubramanian, V. (2018). Environmental deformations dynamically shift the grid cell spatial metric. Elife, 7, e38169

8) Corner cells - please give an intuitive explanation (not just showing this in simulation) why the SR model leads to corner cells. Please also check the range of possible BVC models - maybe corner cells emerge naturally in BVC models (for example by a larger integration of walls within the boundary vector due to increased curvature)?

9) I am not sure the RSA is the best measure for similarity. Why is it better than smoothing the matrix and then simply correlating the population vectors generated from the rate maps? Also - this method does not take into account similarities by symmetry. i.e. things that are symmetrical may be considered more similar if they look the same. Also - perhaps the similarity could be easier to explain by a similar spatial frequency characteristic of the rate maps than by this RSA analysis?

10) line 328 - a technical point - it is better to systematically vary log(1-gamma) that varying gamma itself, as you are most interested in the values approaching 1.
Related to this - the optimal values cannot be chosen at the end of the ranges, as it could be that there are better values below 0.9, or at close values to 1 (thus using log(1-gamma) would be better)

(Remarks on code availability)
I did not review the code.

Reviewer #3

(Remarks to the Author)
The study titled "Subicular spatial codes arise from predictive mapping" by Benneth and de Cothi et al. explores the

application of the successor representation (SR) framework to provide a unified account of the variable spatial firing patterns of principal cells in the subiculum. Previous work has successfully deployed the successor representation framework to describe – and interpret – the firing pattern of hippocampal CA1/CA3 principal neurons in the context of a predictive map. Within this framework, firing of "place" cells code for expected future occupancy of other locations. Here, the authors build on the previous work related to the successor representation model of hippocampal neurons and apply and expand the framework to describe spatial firing patterns of subicular neurons. The authors use detailed simulations and similarity analyses to experimental data from mice and rats and convincingly demonstrate that subicular firing patterns are consistent with representing a successor representation of future spatial states if the time horizon – or the discount parameter gamma – is chosen appropriately. Specifically, they demonstrate that the successor feature model outperforms the boundary vector cell model in predicting the firing patterns of boundary vector cells in the subiculum. In addition, the successor feature model provides an explanation for the diverse firing patterns observed in the subiculum and generates corner cells, a recently characterized subicular subclass of functionally defined cell types. Applying the successor feature model to the subiculum is innovative and expected to be impactful, as it will likely catalyze future computational and experimental research in the field of spatial navigation and in studies of the subiculum in particular. The manuscript is well written and the results support the conclusions. Overall, I am very enthusiastic about this manuscript. I only have one comment as detailed below.

Major comment:
It appears that the authors chose the discount parameter gamma to optimize the similarity between subicular firing fields recorded in experimental data and the firing fields in the simulations. One may ask then, is the choice of this parameter range biologically plausible? Why is the time horizon in subicular representations 16 – 20 s (and 1 – 2 s) in CA1 and not some other value? Does the animal not need representations of expected future states at intermediate time horizons? If yes, then where should we look for those? If not, what is special about these two time horizons, or are the exact discount factors or time horizons dependent on the behavioral or environmental context? Perhaps the authors could discuss in more detail – or state the lack of current knowledge/understanding – for why a predictive map with a time horizon of 16 – 20 s may be useful for informing animal behavior in the context of the successor representation framework.

(Remarks on code availability)
The code is well documented and is a usable resource for the community.

Version 1:

Reviewer comments:

Reviewer #1

(Remarks to the Author)
The authors have provided thorough responses to my comments. The revised manuscript is substantially strengthened by the addition of the new simulations (hierarchical cascade analysis & field-skew effect), analyses (visualization of contributing basis features), and clarifications of interpretation (discussion of predictive horizons and the limitations of the current behavioural paradigm). The authors have adequately addressed my major concerns and I believe that the revised manuscript makes valuable contributions that are worthy of publication.

(Remarks on code availability)

Reviewer #2

(Remarks to the Author)
I went through the response of the authors and believe that they have adequately answered all of my raised concerns.

(Remarks on code availability)

Reviewer #3

(Remarks to the Author)
The authors addressed my previous comment with additional discussion. The revised manuscript has improved. It provides a quite unique and highly innovative explanation for multiple subicular cell types, including corner cells, and is likely to have significant impact on future studies of spatial navigation and subicular function. The manuscript is well written, the conclusions are well supported, and overall the study is highly compelling.

(Remarks on code availability)

Open Access This Peer Review File is licensed under a Creative Commons Attribution 4.0 International License, which permits use, sharing, adaptation, distribution and reproduction in any medium or format, as long as you give appropriate credit to the original author(s) and the source, provide a link to the Creative Commons license, and indicate if changes were made.
In cases where reviewers are anonymous, credit should be given to 'Anonymous Referee' and the source.
The images or other third party material in this Peer Review File are included in the article's Creative Commons license, unless indicated otherwise in a credit line to the material. If material is not included in the article's Creative Commons license and your intended use is not permitted by statutory regulation or exceeds the permitted use, you will need to obtain permission directly from the copyright holder.
To view a copy of this license, visit https://creativecommons.org/licenses/by/4.0/

Response to Reviewer 1

We thank the reviewer for their detailed and constructive feedback. Their comments have prompted several important additions that we believe substantially strengthen the manuscript.

1. Fig. 2f reports only the differences between models and subiculum, without giving the actual proportion of boundary cells in the subicular data. Please report these the empirical values.

We thank the reviewer for highlighting this omission. We have revised Fig. 2f to directly report the proportion of cells classified as boundary cells in each population. In the empirical subicular data, 528/1285 cells (41.1%) were classified as boundary cells using the Muessig et al. procedure. The successor feature model produced the closest match to this proportion (427/1200, 35.6%), while the place cell control substantially underestimated it (287/1200, 13.9%). Because boundary vector cell model responses are boundary-tuned by construction, all units are classified as boundary cells (1200/1200, 100%). The main text and figure caption have been updated accordingly:

> *"Following Muessig et al.[21], we classified cells as boundary vector cells by fitting the boundary vector cell model across a range of distance and direction parameters and identifying cells whose best-fitting correlation exceeded r > 0.7. We then compared the proportion of boundary cells in the empirical subicular recordings (n=1285 cells) to three models (Fig. 2f): the successor feature model (n=1200, trained on the longest rodent trajectory from each dataset), a Gaussian place cell control (n=1200), and the boundary vector cell model itself (n=3120). The successor feature model produced the closest match to the empirical proportion (SF: 427/1200, 35.6%; subiculum: 528/1285, 41.1%), while the place cell control substantially underestimated it (287/1200, 13.9%). Because boundary vector cell model responses are by construction boundary-tuned, all units were classified as boundary vector cells (1200/1200, 100%), far exceeding the proportion observed experimentally."*

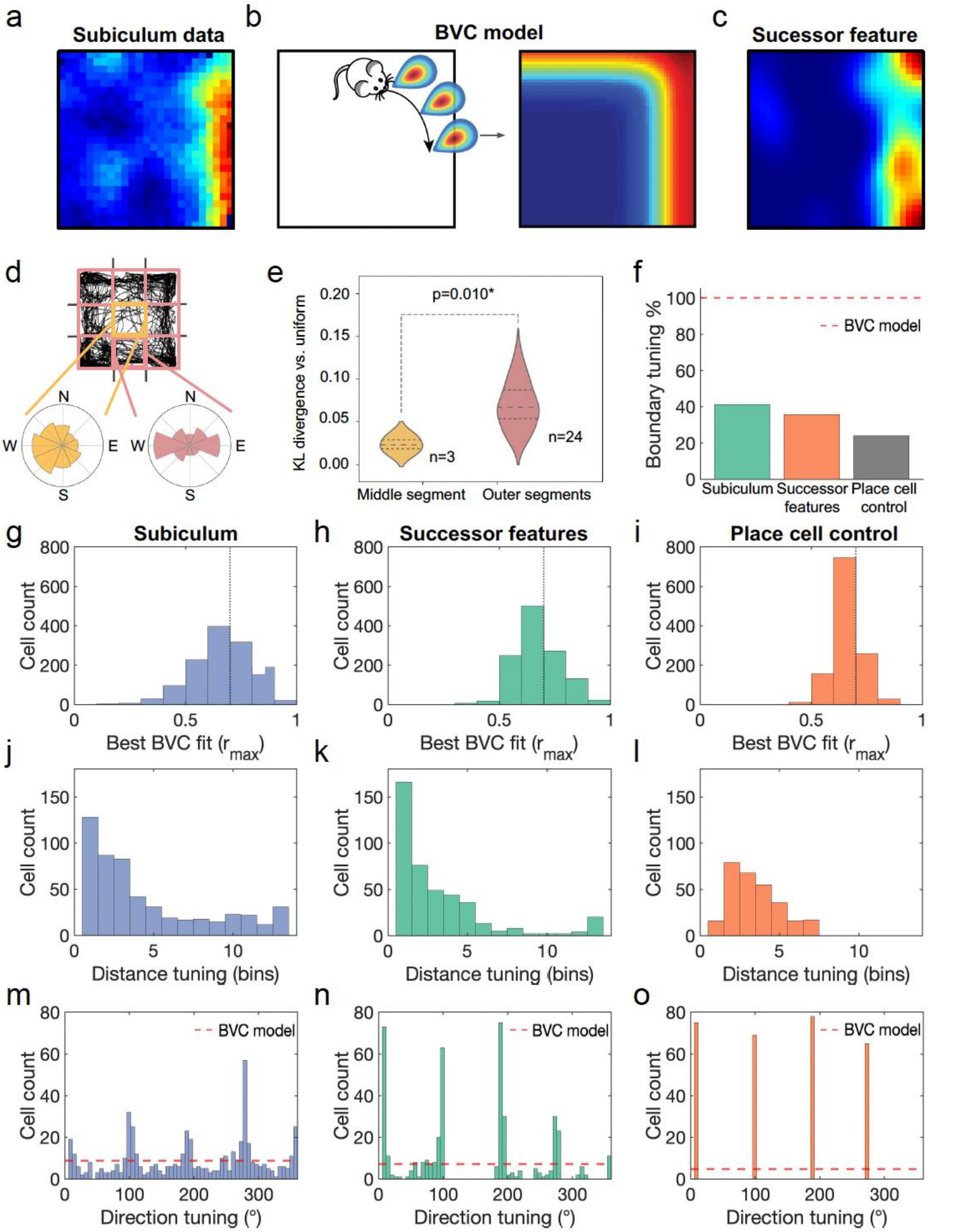

Updated Fig. 2 Successor features produce subicular boundary representations. (**a**) Boundary-responsive cells in rodent subiculum have previously been explained by (**b**) the boundary vector cell model as having receptive fields tuned to environmental boundaries at certain directions and distances. Conversely, the successor feature model (**c**) predicts that behavioural biases near walls produce similar boundary responses in the resulting successor features. This is due to (**d**) trajectory heading directions being more heterogeneous in the central portion of the environment than at the perimeter adjacent to walls, where trajectories are constrained both directionally and by anxiety behaviours such as thigmotaxis, measured by (**e**) the Kullback-Leibler divergence of trajectory headings vs uniform distribution (pairwise t-test middle vs outer segments, using the longest distance trajectory from each dataset, $t(2)=-9.97$, $p=0.010$). (**f**) Proportion of cells classified as boundary cells using the Muessig et al. procedure[21] for subicular data (Sub; 528/1285, 41.1%), successor features (SF; 427/1200, 35.6%), Gaussian place cell control (PC; 287/1200, 13.9%), and the boundary vector cell model (BVC; 1200/1200, 100%; dotted line). Because BVC model responses are by construction boundary-tuned, all units are classified as boundary cells (red dashed line). The successor feature model most

closely matches the empirical subicular proportion (5.5% discrepancy, compared to 27.2% for the place cell control). Fitting the boundary vector cell model to each population revealed that model fits were most similar between (g) the subiculum data and (h) the successor feature model (t-test following Fisher z-transform: t(2483)=0.057, p=0.955; dotted line at rmax=0.7 indicates threshold criteria for a boundary cell), producing significantly better fits than (i) the place cell control model (SUB vs PC, t(2483)=5.67, p<0.001; SF vs PC, t(2483)=6.54, p<0.001). (**j**) Subicular boundary cells were found to over-represent boundary responses proximal to environmental walls, better matching (**k**) the successor feature model than (**l**) the place cell control (pairwise t-test on absolute probability density residuals between SUB-SF vs SUB-PC, t(12)=3.672, p=0.003). Crucially, (**m**) vectorial responses in subiculum were clustered around directions orthogonal to the walls in a square arena (quadrupled, wrapped directional tunings to test four-fold symmetrical clustering: Rayleigh test for non-uniformity, v=90.9, p<0.001), as predicted by (**n**) the successor feature model (quadrupled, wrapped direction tunings, Rayleigh test for non-uniformity, v=232.7, p<0.001; Watson-Williams test of circular means, SUB vs SF, $F_{1,953}$=0.541, p=0.462), while the canonical boundary vector cell model predicts evenly-distributed tunings[18,19] (dashed line) and (**o**) the place cell control model only detects vectorial tunings perpendicular and slightly distal to boundaries. *p<0.05; **p<0.01;***p<0.001.

2. Please add a visualization of the spatial distribution of the basis (place-cell) fields that served as cumulants for the subset of successor feature units that were classified as boundary cells and corner cells. Ideally, this visualization should be done separately for each directional mode of the boundary cell subpopulation and for each corner preference of the corner cell subpopulation. Similarly, in the barrier-duplication analyses, please provide visualizations of the spatial distribution of the basis (cumulant) place-cell fields for duplicating vs. non-duplicating SF (subicular) units.

We agree that visualising the relationship between input basis features and emergent successor feature properties is important for building intuition around the model's predictions. We have added Supplementary Fig. 4, which displays the rate maps and firing field centres of the top contributing basis features for exemplar successor features of each functional class.

For successor features classified as boundary cells, the highest-weighted basis features have firing fields concentrated along the corresponding boundary (Supplementary Fig. 4, columns 1–2), confirming that boundary tuning arises from the spatial distribution of inputs near walls. Corner-classified successor features, by contrast, draw on basis features with more broadly distributed fields — including those centred along boundary midpoints, where animals are likely to travel towards either adjacent corner (Supplementary Fig. 4, column 3). The trajectory-dependent integration of these dispersed inputs produces the characteristic multi-lobed corner response. For the barrier insertion analysis, whether a successor feature duplicates upon barrier insertion depends on the proportion of its highest-weighted basis features that themselves develop duplicated fields in response to the barrier. Successor features whose dominant inputs include a high proportion of duplicating basis features — typically those with fields in the region adjacent to the barrier — duplicate accordingly (Supplementary Fig 4, columns 4–5), while those drawing primarily on non-duplicating basis features do not (Supplementary Fig. 4, columns 6–7).

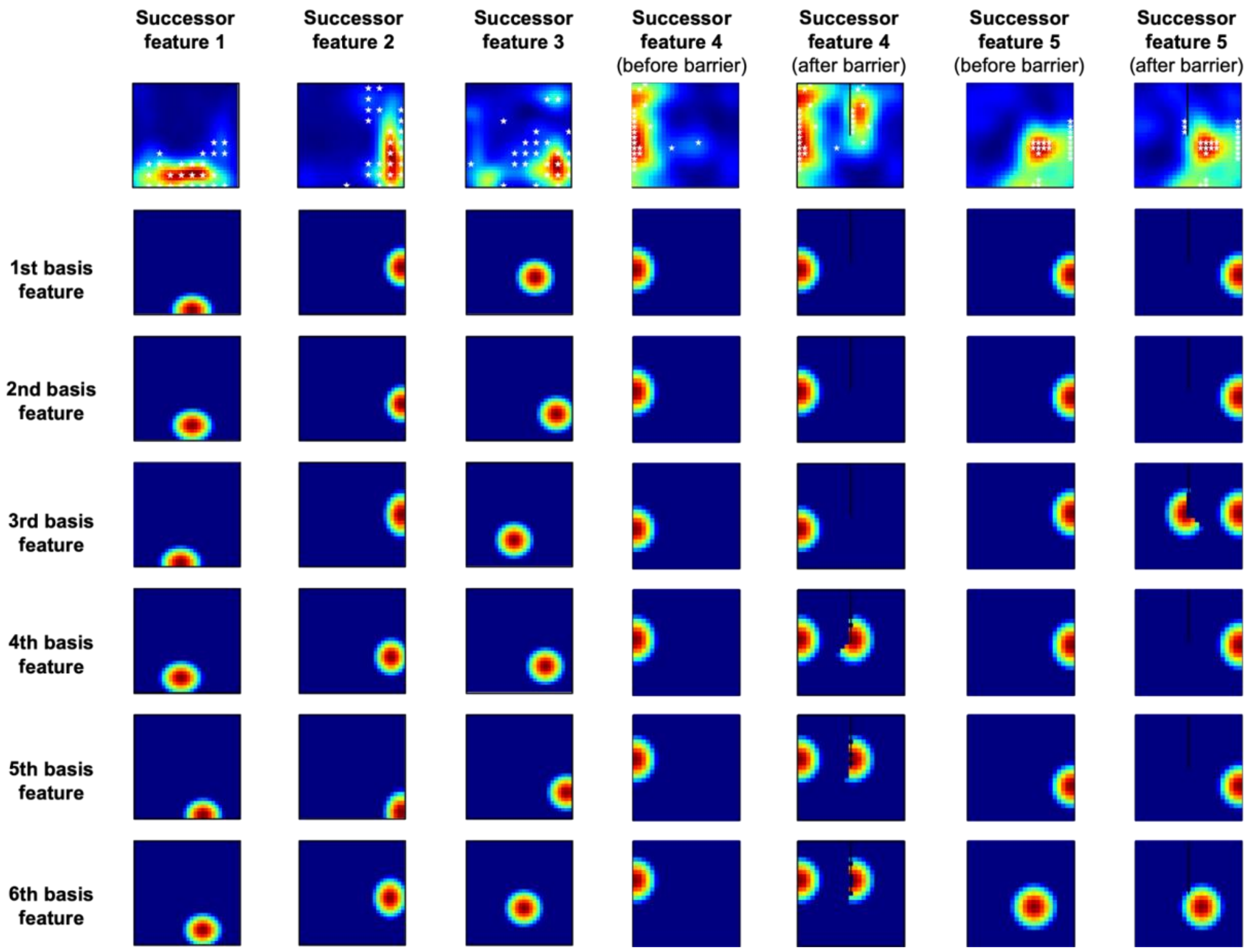

New Supplementary Fig. 4 Spatial distribution of top contributing basis features to a range of successor features. Example spatial tunings of successor features (top row, with white stars denoting field centres for largest contributing basis features) and their largest contributing basis features (columns – ordered by weight). Successor features 1 and 2 are classified as boundary responsive cells with tunings to the south and east wall respectively, with top contributing basis features primarily spanning these regions. Successor feature 3 is classified as a corner cell, as a result of top contributing basis features clustering in corner locations. Successor feature 4 is classified as a boundary responsive cell tuned to the west wall, that duplicates upon barrier insertion because a high proportion of its top contributing basis features themselves duplicate in response to the barrier. Successor feature 5, whose top contributing basis features largely do not duplicate, does not show this effect.

3. Fig. 4 shows that subicular fields shift their centre-of-mass against the dominant axis of travel across consecutive trials. This is argued to be consistent with the SR account, but there is no companion simulation. If these data are to be cited as support for the model, then an SR simulation that reproduces the effect should be provided.

We thank the reviewer for this suggestion. We have now included a companion simulation demonstrating that successor features trained on rodent trajectories reproduce the observed centre-of-mass shift (Supplementary Fig. 3). We generated boundary-tuned successor features from consecutive segments of the training trajectory and quantified the skew of each field relative to the animal's dominant axis of travel. Consistent with the experimental data (Spearman's $r(145) = -0.22$, $p < 0.01$), simulated successor features showed a significant negative relationship between behavioural skew and change in field skew (Spearman's $r(64) = -0.21$, $p < 0.05$; Pearson's $r = -0.28$, $p < 0.05$), confirming that the backward shift in centre-of-mass emerges naturally from the successor representation framework without additional parameter tuning.

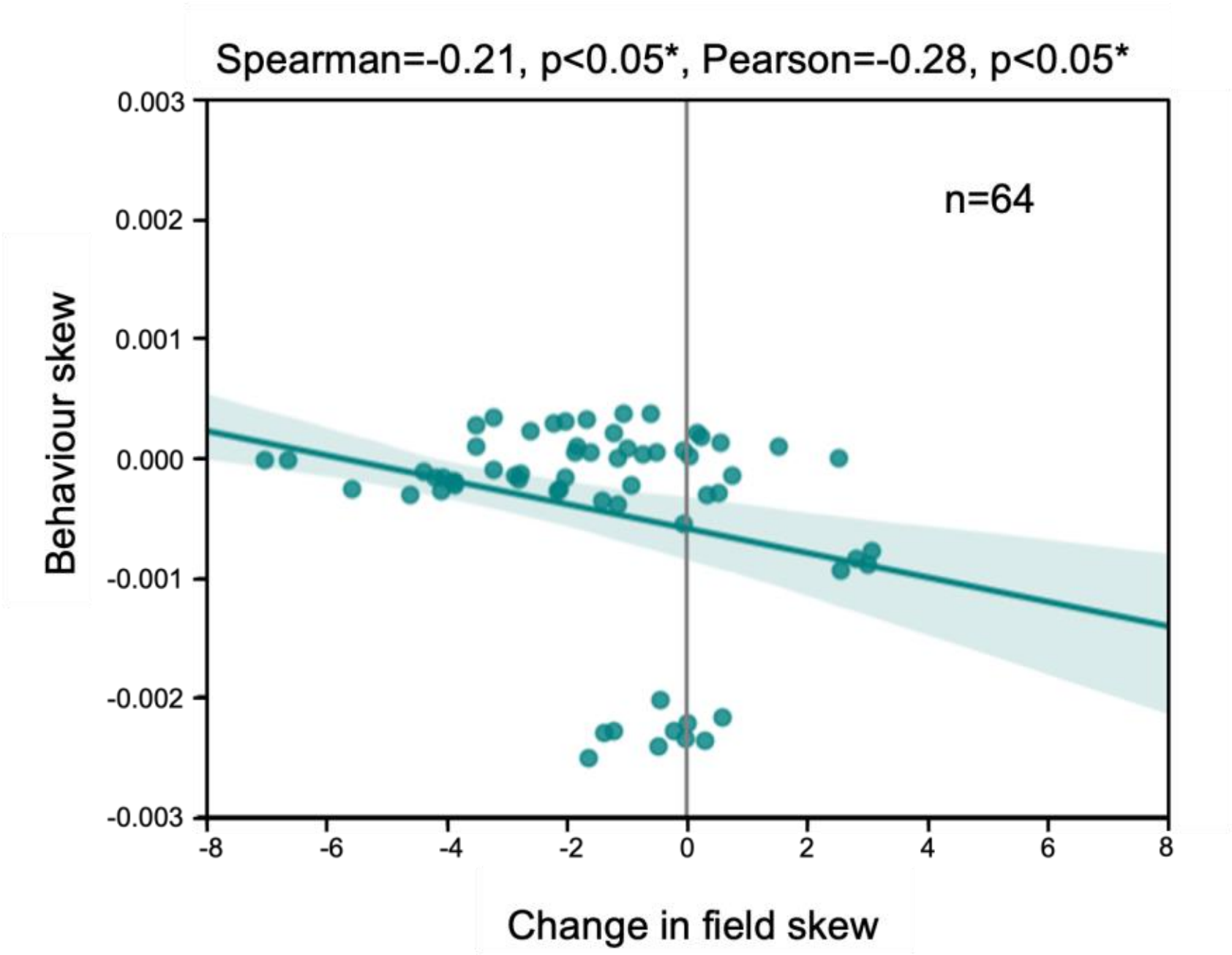

New Supplementary Fig. 3 Successor features skew against the dominant axis of an animal's trajectory in a 2D environment. As previously reported in 1D environments, boundary-tuned successor features that are formed from the two longest distance consecutive rodent trajectories skew negatively against the animal's dominant axis of travel parallel to the field-responsive wall.

4. Figure 7 shows that small γ yields SFs resembling the original basis. While this is useful as a sanity check, it seems somewhat tautological that small γ will transform the basis set less than large γ. A more probative test of the paper's interesting claims about hierarchical processing would be to simulate a two-stage cascade (φ → small γ SF φ → large γ SF) and compare the final layer SFs to the current one-stage setup (φ → large γ SF).

We thank the reviewer for this suggested sanity check. We have implemented a two-stage cascade model in which basis features are first transformed at γ=0.9 (analogous to CA1 processing), and the resulting successor features serve as input to a second transformation at γ=0.995 (analogous to subicular processing). The second-stage successor features retain and amplify the spatial coding properties that emerge in the single-stage model, with an increased proportion of cells classified as corner cells (18% vs 5–6%) and boundary vector cells (72% vs 36%). This demonstrates that the hierarchical architecture produces boundary and corner representations robustly, and that the predictive structure encoded at the first stage is further elaborated — rather than simply overwritten — by the second transformation. We have added the following text to the results, which are presented in Fig. 7d below:

> *"To test whether these spatial coding properties could emerge through a hierarchical architecture, we implemented a two-stage cascade in which basis features were first transformed at γ=0.9, and the resulting successor features served as input to a second transformation at γ=0.995 (Fig. 7d). The second-stage successor features produced a greater proportion of corner cells (18%) and boundary vector cells (72%) than the single-stage model (5–6% and 36% respectively), demonstrating that boundary and corner representations emerge*

robustly from the hierarchical architecture, with the predictive structure encoded at the first stage being further elaborated by the second transformation. These results are not contingent on synthetic basis features: successor features built on 67 simultaneously recorded CA1 place cells[21,60,61] still produce corner cells (4.5%) and boundary vector cells (74%; Supplementary Fig. 5). The higher proportion of boundary cells likely reflects the sparse, non-uniform coverage provided by this small population (n=67), which over-samples near-wall fields relative to the larger synthetic basis set."

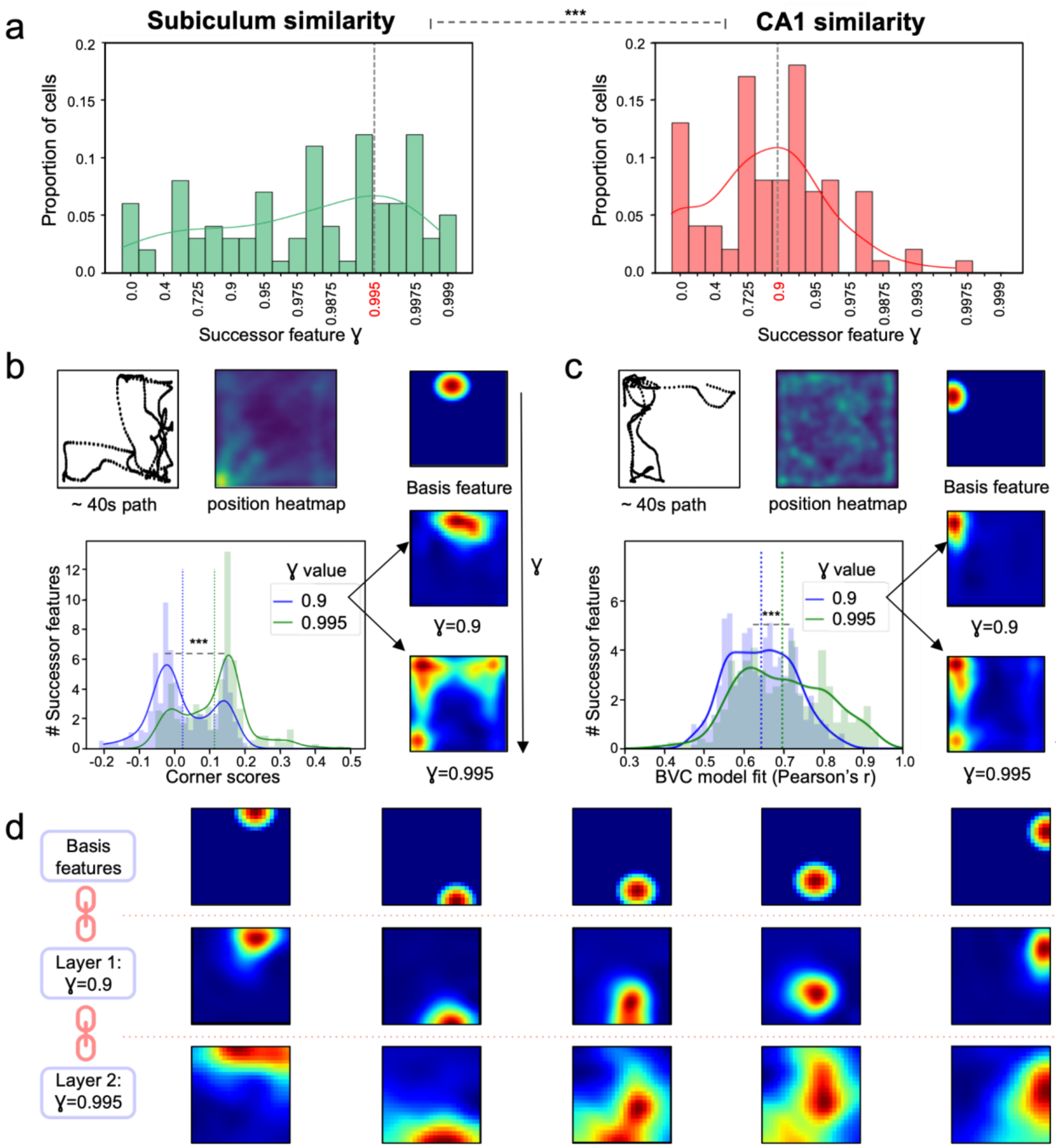

Updated Fig. 7 Distinct temporal horizons capture subicular and hippocampal representations. (**a**) Distribution of best-fit γ values for subicular (left) and CA1 (right) cell responses; kernel density estimate (KDE) shown as solid line, vertical line indicates KDE maximum. Subicular cells were best fit by γ ≈ 0.995 (temporal horizon ~20 s), while CA1 cells were best fit by γ ≈ 0.9 (temporal horizon ~1 s). (**b**) Corner representations emerge with extended temporal horizon. Top: trajectory (~40 s shown) and position heatmap over full 20 minute trial illustrating corner-dwelling behaviour. Right: a corner-proximal basis feature at γ = 0.9 (CA1-like) and γ = 0.995 (subicular-like), demonstrating how extended temporal integration bridges multiple corner locations. Bottom: distribution of corner scores across successor

features shifts towards larger values with increasing γ; vertical lines indicate mean values. (**c**) Boundary representations emerge with extended temporal horizon. Top: trajectory (~40 s shown) and heatmap over full 15 minute trial demonstrating thigmotaxic behaviour. Right: a boundary-proximal basis feature transforms into an elongated boundary response at $\gamma = 0.995$ (~20 s predictive horizon). Bottom: distribution of boundary vector cell model fits (Pearson's r) shifts towards higher values with increasing γ; vertical lines indicate mean values. *$p < 0.05$; **$p < 0.01$; ***$p < 0.001$. (**d**) Two-stage cascade model in which basis features are first transformed at $\gamma = 0.9$ (Layer 1, analogous to CA1) and the resulting successor features then serve as input to a second transformation at $\gamma = 0.995$ (Layer 2, analogous to subiculum). Layer 2 produces a substantially greater proportion of corner cells (18% vs 0%) and boundary vector cells (72% vs 7%) than Layer 1, and amplifies both cell types relative to the single-stage model (5–6% corner, 36% BVC at $\gamma = 0.995$), indicating that the second stage elaborates predictive structure already present in the first.

5. The results are interpreted as showing that subiculum encodes a longer predictive horizon than CA1 (~20 s vs. ~1–2 s). However, the open-field foraging paradigm incorporates a stable policy with constant state values; there is no need to revalue specific states for decision-making. SRs were originally proposed to enable flexible revaluation in dynamic environments with changing reward contingencies. Thus, the apparent fit of subiculum to long predictive horizons may partly reflect the behavioural context rather than general coding principles. A stronger test would use an experimental/simulation paradigm in which the SR can perform its intended function of rapid revaluation with slower, policy-dependent SR updating. Please consider in simulations or discussion how task demands might shift the effective predictive horizon.

The reviewer raises an important point. Our primary analyses use open-field foraging data where the behavioural policy is relatively stable and well-learned. This permits direct comparison between CA1 and subicular data, because recordings are sampled under the same behavioural policy. The successor representation was originally formulated to separate predictive structure from reward values (Dayan, 1993, ref [44]), enabling rapid revaluation when contingencies change. Our results demonstrate that this predictive structure alone — independent of reward — is sufficient to account for the heterogeneous spatial responses in subiculum, and we identify the dynamics of subicular updating under new behavioural policies as a key direction for future work. To our knowledge, no existing data allow a direct comparison of subicular and CA1 activity under changing task demands, thus how these representations adapt remains an important open question that we have now addressed in a new paragraph in the Discussion (alongside a related point by Reviewer 3):

> *"These two timescales should not, however, be taken as the only predictive horizons present in the hippocampal formation. Individual cells within both CA1 and subiculum span a range of optimal γ values (Fig. 7a), indicating that each region expresses a distribution of predictive horizons rather than a single fixed timescale. More broadly, the well-established dorso-ventral increase in place field size can be understood as a gradient of predictive timescales[79], with ventral hippocampal regions encoding longer-horizon successor representations than dorsal. Our recordings, drawn from dorsal hippocampus and subiculum, capture two points along what may be a continuous spectrum. Importantly, the central finding — that subicular representations are best approximated by successor representations with longer temporal horizons than those fitting CA1 — is a relative observation that is robust across our analyses and does not depend on the absolute value of γ. Whether the relative ordering of predictive horizons between CA1 and subiculum is maintained under goal-directed or changing behavioural policies — and whether the absolute timescales shift with task demands — represents a key direction for future work. The successor representation was originally formulated to enable rapid revaluation when reward contingencies change, by separating the*

predictive structure of the environment from current reward values[44]. Our findings demonstrate that this predictive structure alone — independent of reward — is sufficient to account for the heterogeneous spatial responses observed in subiculum. An important open question is how the temporal hierarchy we identify here responds when animals must flexibly adapt their behaviour under changing task demands. In particular, the dynamics of subicular updating under new behavioural policies — and whether the relative ordering of predictive horizons between CA1 and subiculum is preserved — remain to be established experimentally."

Response to Reviewer 2

We thank the reviewer for their thoughtful feedback and detailed engagement with our work. Below, we address each point in turn.

1. One major difference between CA1 and subiculum is in the pattern of remapping. How do the authors explain the difference using SR — that subiculum in principle does not remap, while CA1 does? Is there any way to link the successor representation framework to understanding this question?

The difference in remapping behaviour between CA1 and subiculum emerges naturally from the successor representation framework as a consequence of the temporal horizon over which predictions are integrated. At short horizons, each successor feature depends on a small number of neighbouring states and is therefore susceptible to upstream remapping; at long horizons, predictions are integrated over many states, buffering the representation against partial remapping of the input. To demonstrate this directly, we simulated remapping across a range of temporal horizons and show that the degree of successor feature remapping decreases monotonically with increasing γ (Supplementary Fig. 2), providing a natural account for the greater stability of subicular representations relative to CA1 (Sharp, 2006). We have updated the Results and Discussion to reflect this new analysis, and this result also addresses a related point in R2.6 below.

Updated Results:

"Our analyses reveal two key properties of successor feature boundary responses that align with empirical observations. First, these responses show resistance to place cell remapping: while remapping in the place cell basis can propagate to some successor features (Fig. 3a), the boundary code largely maintains stability despite moderate upstream changes (Fig. 3b). This stability might explain the rapid formation and persistence of boundary responses in novel environments[8,20,24], with the degree of remapping resistance scaling monotonically with the temporal horizon of the predictive map (Supplementary Fig. 2)."

Updated Discussion:

"Notably, our remapping analyses (Fig. 3, Supplementary Fig. 2) reveal that successor representation boundary responses exhibit some resistance to place cell remapping, in a manner that scales with the predictive horizon. This potentially accounts for the rapid formation and stability of boundary responses observed in novel environments[24]."

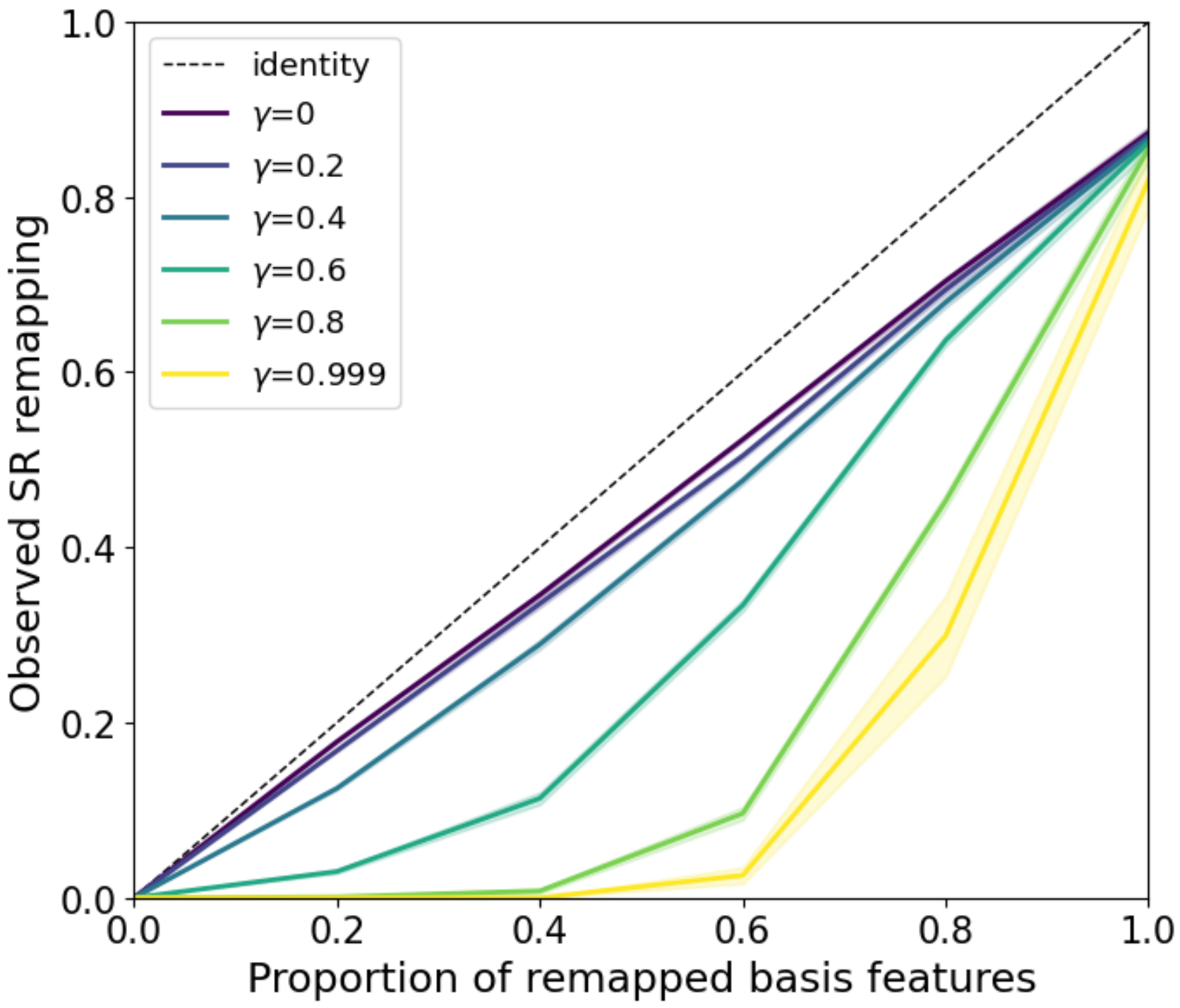

New Supplementary Fig. 2. Resistance to remapping scales with predictive temporal horizon. Observed successor feature remapping as a function of the proportion of remapped basis features, across a range of temporal horizons (γ = 0 to 0.999). At short horizons, successor feature remapping closely tracks basis feature remapping (dashed identity line). As the temporal horizon increases, successor features become progressively more resistant to upstream perturbations, with long-horizon representations (γ = 0.999) substantially buffered against partial remapping of the input. Shaded regions indicate standard error of the mean calculated across 10 random seeds.

2. Another textual issue (e.g. line 62) — to claim that SR is a class of a predictive algorithm is not completely precise, as it is only a predictor of action, while not being a predictor, for example, of the sensory input. I would make this distinction in the text.

We thank the reviewer for this point. We note that the citations used alongside the 'predictive algorithms' reference (line 62 as quoted by the reviewer) refer broadly to a family of computational frameworks that use some form of prediction to explain hippocampal spatial representations — encompassing models of sensory prediction (e.g. Whittington et al., 2020; Uria et al., 2022), sequential learning (e.g. Levenstein et al 2024) as well as those that predict future state occupancy (e.g. Stachenfeld et al., 2017). The successor representation specifically predicts expected future state visitation under a given behavioural policy, rather than sensory input per se. We have clarified this distinction in the text:

> *"Recently, predictive algorithms—encompassing models of sensory prediction[40,41,77], sequential learning[43], and expected future state occupancy[39,42]—have emerged as a powerful framework for understanding hippocampal function and the generation of neural responses in associated regions[38–43]."*

3. Fig 1a — how does the choice of the basis feature affect the results? Instead of taking synthetic basis features, why not use rate maps of real place cells?

We thank the reviewer for raising this point. The choice to use synthetic basis features was motivated by two practical considerations. First, it provides full control over the spatial statistics of the basis population, allowing us to systematically investigate how known properties of place cells (e.g., wall-proximity effects on field size, boundary-dependent elongation) influence the emergent successor features. Second, limited sample sizes and recording biases inherent in real electrophysiological data confound the successor representation analysis, which requires a sufficient density of basis features that uniformly sample the environment to produce well-structured successor features.

A small population of CA1 cells (353 ratemaps from 3 rats), however, were also recorded in the Muessig et al. (2024) experiment, and 67 of these cells were recorded over two consecutive trials. We find the successor features generated from these CA1 recordings and animal behaviour produce boundary and corner cell responses (74% and 4.5% respectively). The higher proportion of boundary cells likely reflects the sparse, non-uniform coverage provided by this small population (n=67), which over-samples near-wall fields relative to the larger synthetic basis set. We have now included this analysis in Supplementary Fig. 5 and display more of the resultant successor features below in Figure R1.

New addition to the results:

> *"These results are not contingent on synthetic basis features: successor features built on 67 simultaneously recorded CA1 place cells[21,60,61] still produce corner cells (4.5%) and boundary vector cells (74%; Supplementary Fig. 5, see Methods). The higher proportion of boundary cells likely reflects the sparse, non-uniform coverage provided by this small population (n=67), which over-samples near-wall fields relative to the larger synthetic basis set."*

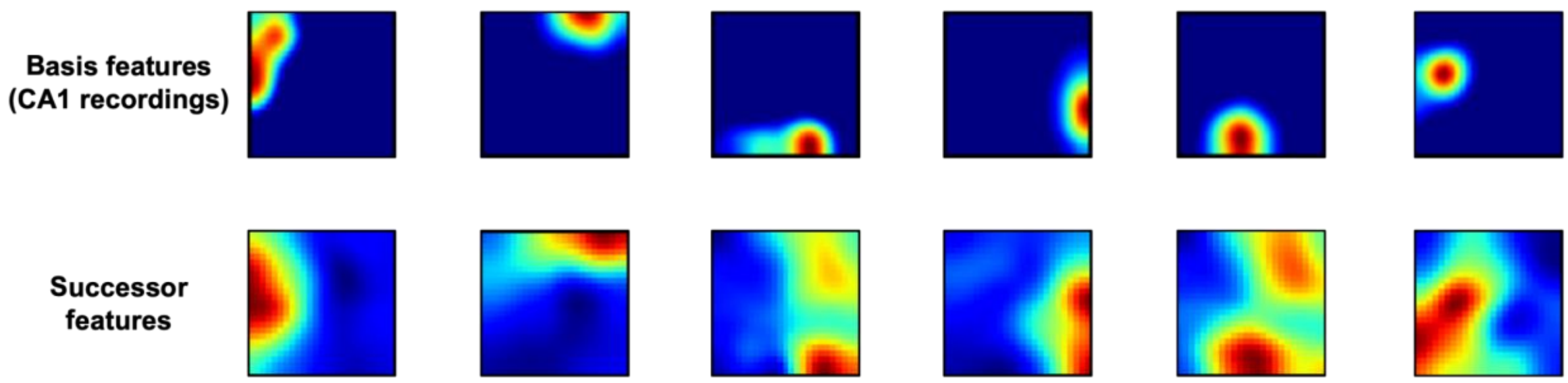

New Supplementary Fig. 5 Single population of successor features built on real CA1 recordings. For one environment (Muessig and colleagues[21,60,61]), 67 CA1 place cells were recorded over two consecutive trials, and we use these with their longest distance rodent trajectory to build a single population of 67 successor features, which reproduce the topology of successor features built on biologically plausible basis features. 4.5% of these resulting successor features are classified as corner cells and 74% are classified as boundary vector cells.

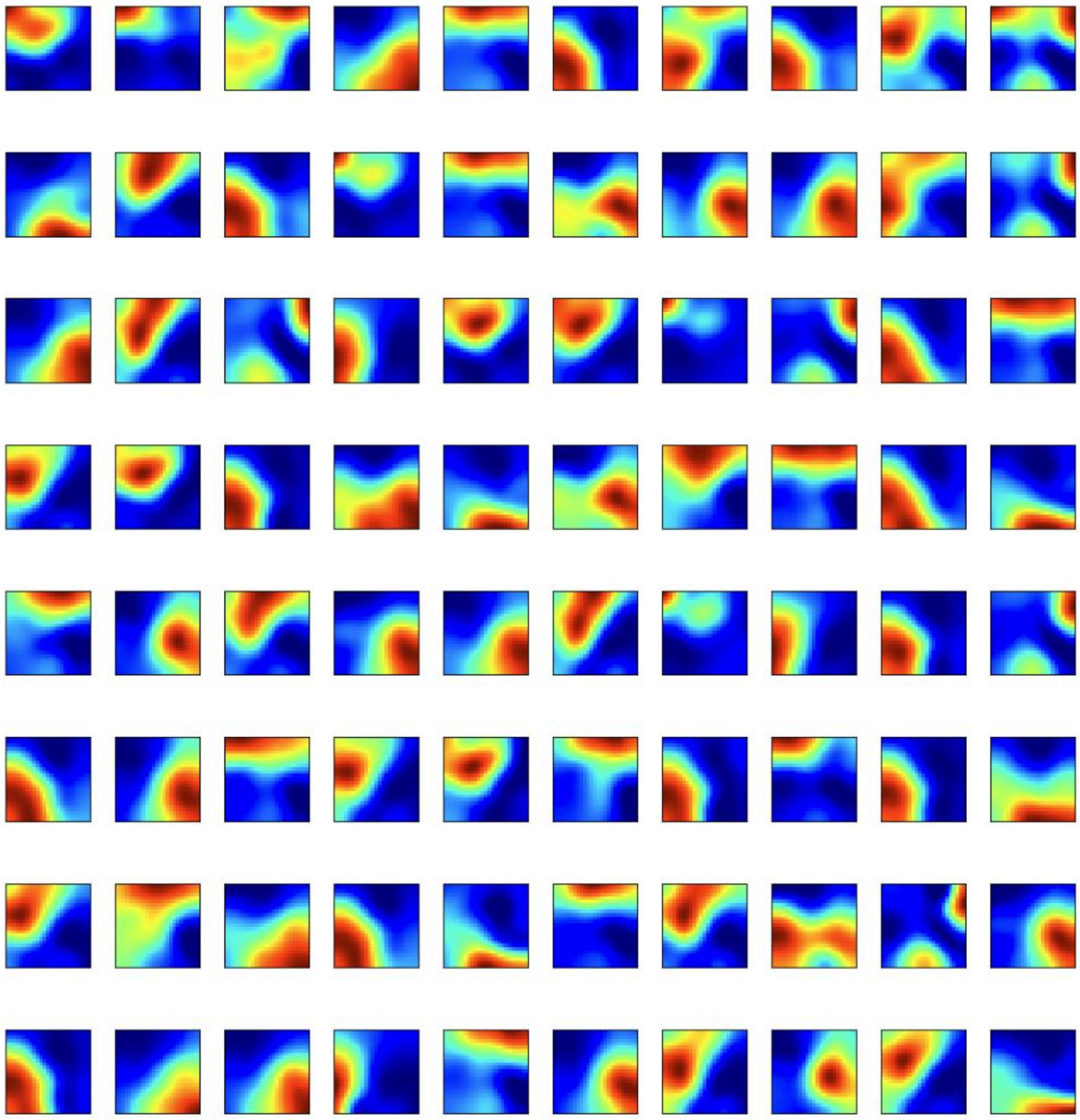

Fig.R1 Larger sample of successor features generated from CA1 basis features.

4. Figure 2 — Using the procedure of Muessig et al. is compelling, however I do not believe this is optimal in this case, because if the question is to compare BVC models to SR models, then these are the models that should be compared. What should be done is to create a whole set of SR models (with different discounts into the future) and a whole set of different BVC models (with different radii and opening angle) and then test these on the data, preferably using a training set and then testing using a test set.

We thank the reviewer for this suggestion and appreciate the logic of a direct cross-validated model comparison. However, on reflection we believe this framing does not quite match the question our analysis is designed to address. The goal of Figure 2 is not to determine whether the successor representation fits individual subicular cells better than the boundary vector cell model — a question for which the reviewer's proposed approach would be appropriate. Rather, Figure 2 asks whether the population-level statistics of the successor feature model match those observed in subiculum: specifically, whether the proportion of cells classified as boundary vector cells under an established, independent classification procedure resembles the empirical proportion. We appreciate that this distinction was not sufficiently clear in the original manuscript and have revised both the text and Figure 2f, which now displays the absolute proportion of cells classified as BVCs for each population: subicular data (528/1285, 41.1%), successor features (427/1200, 35.6%), place cell control (287/1200,

13.9%). Because responses generated by the boundary vector cell model are by construction boundary-tuned, all units are inevitably classified as BVCs (1200/1200, 100%; shown as a dotted line in Fig. 2f). This makes clear that the successor feature model produces BVCs at a rate closely matching the experimental data, while the place cell control substantially underestimates and the boundary vector cell model, by definition, overestimates the observed proportion. We have also revised the accompanying text to clarify the logic of this comparison.

Beyond this population-level comparison, we note that Figure 2g–o does provide a detailed quantitative comparison between the successor feature model and the boundary vector cell model fits. The distribution of best-fitting BVC model correlations is statistically indistinguishable between subicular data and successor features (Fig. 2g,h; p=0.955), while both significantly exceed the place cell control (Fig. 2i; p<0.001). Furthermore, the successor feature model matches the subicular data in the distribution of preferred boundary distances (Fig. 2j–l) and the clustering of directional tunings around axes orthogonal to environmental walls (Fig. 2m–o) — a property that the canonical boundary vector cell model, with its uniformly sampled parameters, does not predict.

We note additionally that the Muessig et al. classification procedure has been published and validated against independent electrophysiological datasets, providing a well-established criterion for identifying boundary cells that does not depend on the successor representation framework. Finally, we use a single set of successor representation parameters (γ = 0.995) throughout the manuscript and demonstrate in Figure 7 that this value provides the best fit to the observed subicular data across the full γ range, giving us confidence that the population statistics reported in Figure 2 are not an artefact of parameter selection.

The relevant passage in the results now reads:

> *"We then compared the proportion of boundary cells in the empirical subicular recordings (n=1285 cells) to three models (Fig. 2f): the successor feature model (n=1200, trained on the longest rodent trajectory from each dataset), a Gaussian place cell control (n=1200), and the boundary vector cell model itself (n=3120). The successor feature model produced the closest match to the empirical proportion (SF: 427/1200, 35.6%; subiculum: 528/1285, 41.1%), while the place cell control substantially underestimated it (287/1200, 13.9%). Because boundary vector cell model responses are by construction boundary-tuned, all units were classified as boundary cells (1200/1200, 100%), far exceeding the proportion observed experimentally."*

5. Line 179 — "Conversely, the place cell control model was only matched to perpendicular vectorial tunings" — isn't that an artifact of the way the model was constructed? Please clarify.

We thank the reviewer for this question. The place cell control model is not an arbitrary construction — it is based on the experimentally observed statistics of place cell fields reported by Tanni et al. (2022), including wall-proximity effects on field size and shape. The perpendicular vectorial tunings observed in this control are a consequence of how place cell fields interact with the BVC fitting procedure: place cells located near walls produce ratemaps that, when fit by the BVC model, yield distance tunings close to zero and directional preferences perpendicular to the nearest wall —

effectively, the model is fitting the wall-adjacent edge of the place field rather than detecting genuine boundary coding. We have clarified this in the manuscript:

> *"Conversely, the place cell control model was only matched to perpendicular vectorial tunings (Fig. 2o; Rayleigh test for non-uniformity, v=287.0, p<0.001) and less proximal (Fig. 2l) boundary distances, reflecting the BVC fitting procedure capturing the wall-adjacent edges of near-boundary place fields rather than genuine boundary coding."*

To verify this directly, we ran the boundary tuning classification procedure on a symmetric Gaussian place cell control population. Of these, 82/400 cells (20.5%) exceeded the boundary cell classification threshold (r_max > 0.7), with best-fitting parameters concentrated at short distance tunings and directions perpendicular to the nearest boundary (Fig. R2).

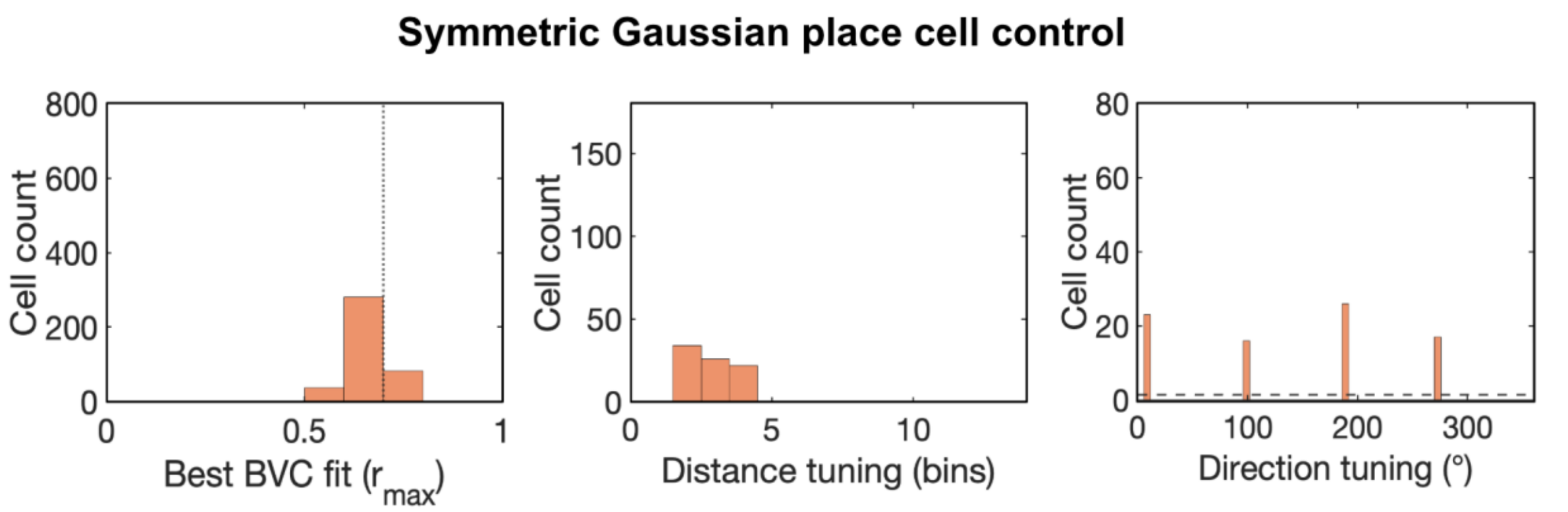

Fig.R2. Boundary vector cell model fits to symmetric Gaussian place cell control. Left: distribution of best-fitting boundary vector cell model correlations (r_max) for 400 symmetric Gaussian place cells; dotted line indicates classification threshold (r_max = 0.7), exceeded by 82/400 cells (20.5%). **Middle**: distribution of best-fitting distance tunings for place cells exceeding threshold, showing clustering at short distances. **Right**: distribution of best-fitting directional tunings for place cells exceeding threshold, showing clustering at 0°, 90°, 180° and 270° — perpendicular to the four walls of the square arena — confirming that the boundary vector cell fitting procedure captures the wall-adjacent edges of near-boundary place fields.

6. Figure 3 — how is the resistance to remapping consistent with the ideas about SR in CA1? Please clarify.

We thank the reviewer for this question, which is closely related to R2.1 above. In our framework, CA1 place cells serve as the basis features (input) to the successor representation computed in subiculum. Remapping of CA1 place cells therefore represents an input perturbation, and the stability of subicular boundary responses (Fig. 3) is the model's predicted output.

The apparent tension with CA1 itself exhibiting SR-like properties is resolved by the difference in temporal horizon. At shorter horizons, each successor feature depends on a smaller number of neighbouring states and is therefore more susceptible to upstream remapping; at longer horizons, predictions are integrated over many states, buffering the representation against partial input remapping. We now demonstrate this directly: remapping resistance scales monotonically with γ across a full range of temporal horizons (Supplementary Fig. 2). See R2.1 for the corresponding manuscript edits.

7. Line 244 — the phenomenon of skew mentioned here was also shown for grid cells by Keinath et al. (2018). Environmental deformations dynamically shift the grid cell spatial metric. eLife, 7, e38169.

We thank the reviewer for drawing our attention to this work. Keinath et al. (2018) demonstrate that grid cell firing patterns undergo dynamic boundary-tethered phase shifts during environmental deformations, driven by border cell inputs. While this is an interesting example of how environmental geometry influences spatial representations, the phenomenon they describe — a translation of grid phase anchored to displaced boundaries — is mechanistically distinct from the behavioural skew we report, in which successor feature centres-of-mass shift against the animal's dominant axis of travel as a consequence of the predictive nature of the representation. We have nonetheless added a reference to Keinath et al. (2018) in our discussion of how environmental geometry shapes spatial representations more broadly (ref 80):

> *"An implication of our work is to propose distinct roles for the two main hippocampal outputs. CA1 appears to transmit a condensed representation of states that are assembled from entorhinal border and grid cell inputs together with path integration and multimodal sensory information, while subiculum represents commonly utilised trajectories afforded by environmental geometry (though see also [39,45,49,80] for models of how geometry influences grid cell representations). This differentiation does not negate prior work proposing that CA1 place fields can be understood within a predictive framework[39,45,54,63]. Indeed, Lee and colleagues[63] demonstrate that when modelling CA1 activity, successor features built on boundary representations (such as those from entorhinal cortex) provide a better account of place cell properties than successor features built on place cell inputs themselves."*

8. Corner cells — please give an intuitive explanation (not just showing this in simulation) why the successor representation model leads to corner cells. Please also check the range of possible BVC models — maybe corner cells emerge naturally in BVC models.

Corner cells emerge in the successor representation model because of the specific pattern of rodent behaviour near corners. Animals dwell disproportionately in corners and, upon leaving, make rapid excursions along adjacent walls to neighbouring corners — where they again pause. Because the successor representation integrates expected future state occupancies, a basis feature located near one corner accumulates predictions encompassing neighbouring corners connected by these stereotyped trajectories. With a sufficiently long temporal horizon ($\gamma = 0.995$, ~20 s), these predictions bridge multiple corner locations, producing multi-lobed firing fields centred on corners. We have incorporated this explanation into the Discussion:

> *"Corner cells emerge naturally from this framework because of pronounced biases in rodent behaviour. Animals dwell disproportionately in corners and, upon leaving, make rapid excursions along adjacent walls to neighbouring corners — where they again pause. Stereotyped wall-running trajectories thus link corners to one another in rapid succession, meaning that occupancy of one corner is strongly predictive of future occupancy at adjacent corners. Because the successor representation integrates expected future state occupancies, a basis feature located near one corner accumulates predictions encompassing neighbouring*

corners connected by these behavioural trajectories. With the extended subicular temporal horizon ($\gamma = 0.995$, ~20 s), these predictions bridge multiple corner locations, producing the characteristic multi-lobed firing fields centred on corners that Sun et al. (2024) identified experimentally."

To address whether corner cells could emerge from the boundary vector cell framework, we systematically varied the angular tuning width from its canonical instantiation (angular tuning width $\sigma_\theta = 0.2$ radians) across $\sigma_\theta = 0.2, 0.4, 0.8$, and 1.6 radians for angular tuning distances (σ_d) = 2, 4, 6, 8, 10cm in a 30x30cm square environment. For each value of σ_θ and σ_d, we generate BVC responses across a range of preferred distance and directional tunings. To apply a shuffling procedure comparable to Sun et al. (2024) — which operates on spike trains rather than rate maps directly — we generated simulated rodent trajectories through each BVC firing field, produced corresponding Poisson-spike trains, and then circularly permuted each spike train relative to position. Corner scores were subsequently recalculated from the reshuffled rate maps following the method of Sun et al. (2024), which notably excludes the penalty term for additional firing fields.

For transparency, we note that the Sun et al. (2024) procedure is asymmetric in its treatment of real and shuffled data: the corner score applied to observed ratemaps includes a penalty term for additional firing fields, whereas the score computed on shuffled ratemaps does not. If the same (unpenalised) corner score is applied to both — departing from Sun et al.'s published procedure — approximately 6% of BVC units with $\sigma_\theta = 1.6$ radians are classified as corner cells, consistent with the expected false-positive rate at the chosen significance threshold.

At canonical and moderate angular tuning widths, individual BVC units produce single-peaked fields near one wall or, at most, elongated fields extending along adjacent walls — rather than the discrete multi-lobed corner-centred pattern observed experimentally. Only at extreme angular tuning widths ($\sigma_\theta = 1.6$ radians), far outside the originally proposed range, did cells begin to emerge whose responses visually resembled the structure of corner cells. However, none of these units passed the Sun et al. (2024) shuffling criterion and none were therefore classified as corner cells, most likely because their firing was insufficiently differentiated between the corner itself and the adjacent wall segments. Ultimately, each BVC response is the product of two unimodal tuning curves (one for distance, one for direction), which constrains the field to a single boundary region; producing genuine corner cells from BVCs would require an additional combinatorial stage that is not part of the standard model.

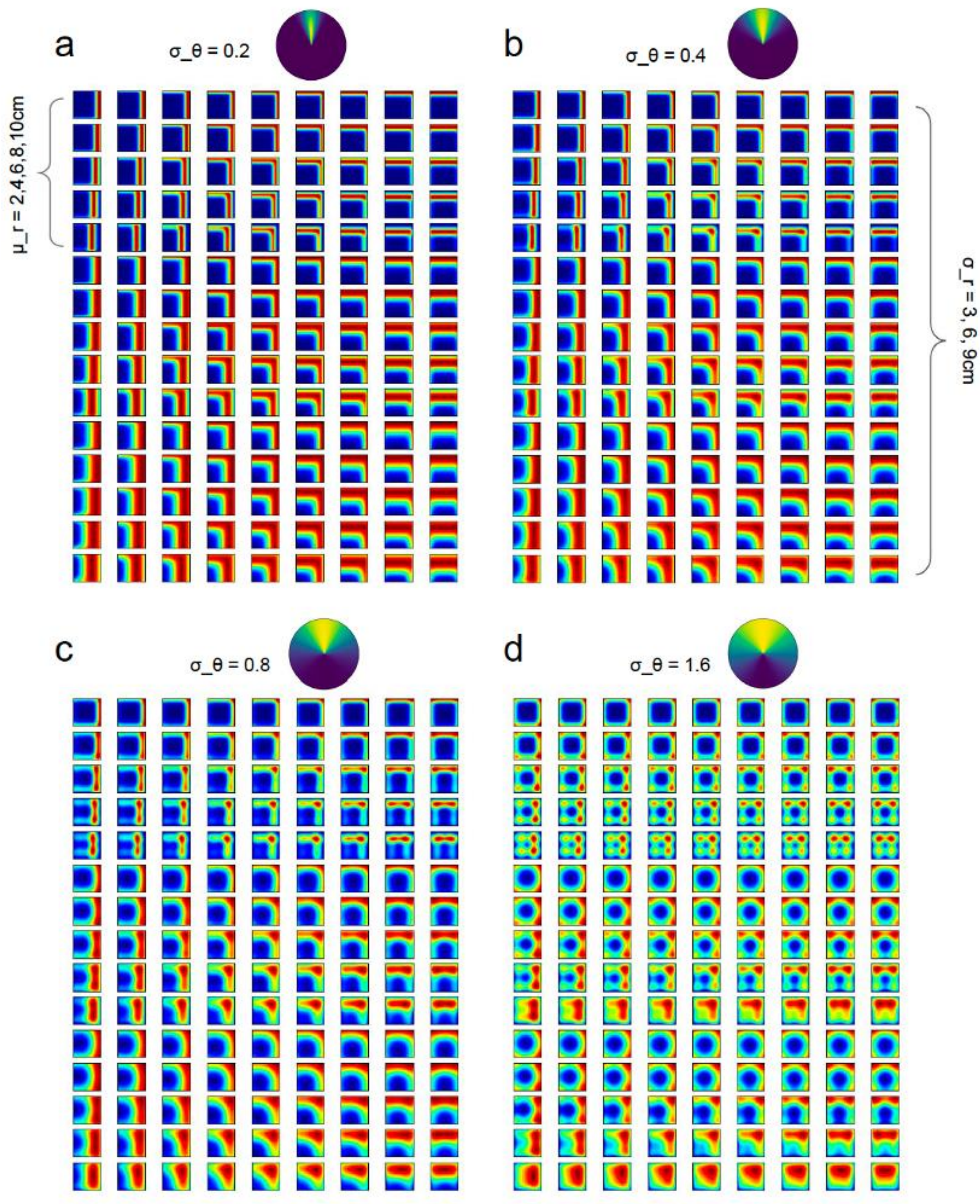

Supplementary Fig. 6 The boundary vector cell model produces fields that resemble multi-lobed corner responses for extreme angular tuning parameters. Boundary vector cell populations and their circular receptive fields covering nine preferred angular directions, five preferred distance tunings (μ_r) and three radial tuning widths (σ_r), shown at four angular tuning widths (σ_θ) of (**a**) 0.2, (**b**) 0.4, (**c**) 0.8 and (**d**) 1.6 radians — i.e. the canonical value used by Hartley et al.[18] (0.2 radians) and 2×, 4× and 8× that value. Fields that resemble multi-lobed corner responses only begin to emerge at σ_θ = 1.6 radians, well outside the parameter range of the original model; however, none of these units passed the shuffling criterion applied by Sun et al.[9] and none were therefore classified as corner cells. Note that at this tuning the model essentially predicts responses against all barriers regardless of their orientation relative to the agent.

9. I am not sure the RSA is the best measure for similarity. Why is it better than smoothing the matrix and then simply correlating the population vectors? Also — this method does not take into account similarities by symmetry. Also — perhaps the similarity could be easier to explain by a similar spatial frequency characteristic of the rate maps?

We thank the reviewer for these methodological questions. We chose RSA following Lee et al. (2023) because it provides a principled framework for comparing population-level representations without requiring cell-to-cell correspondence between model and data — a key requirement when comparing model populations to experimental recordings. We have added this justification to the Results:

> *"To compare population-level representations without requiring cell-to-cell correspondence between model and data, we employed representational similarity analysis (RSA)[62,70] following Lee et al.[63,69]."*

Regarding population vector correlation: this approach would require assumptions about how to align model and experimental populations, which RSA avoids by operating on the pairwise similarity structure within each population.

Regarding symmetry: in our square environment analyses, the 3×3 grid partitioning captures the major symmetry axes of the environment, mitigating the concern that symmetric but functionally equivalent representations would be treated as dissimilar.

Regarding spatial frequency: our analysis already controls for this. The successor features built on real rodent trajectories (SF) and those built on random walk trajectories (SF_RW) are derived from identical basis features and differ only in the training trajectories, yet produce significantly different RSA fits to subicular data (Fig. 6b; SF vs SF_RW: $p=0.022$). This dissociation demonstrates that the superior fit of the trajectory-trained model cannot be attributed to shared spatial frequency content in the basis features.

10. Line 328 — it is better to systematically vary $\log(1-\gamma)$ than γ itself, as you are most interested in values approaching 1. Related to this — the optimal values cannot be chosen at the end of the ranges.

We thank the reviewer for this technical suggestion. We agree that $\log(1-\gamma)$ provides a more uniform sampling of the parameter space near $\gamma=1$, where small changes in γ correspond to large changes in the effective temporal horizon. We have re-run our γ sweep using log-spaced values (γ = 0.0 to 0.999) and confirm that the optimal values for subiculum remain at γ = 0.995 (~20 s temporal horizon) and for CA1 at γ = 0.9 (~1 s temporal horizon). The extended range ensures that both optima fall well within the interior of the search space (updated Fig. 7a).

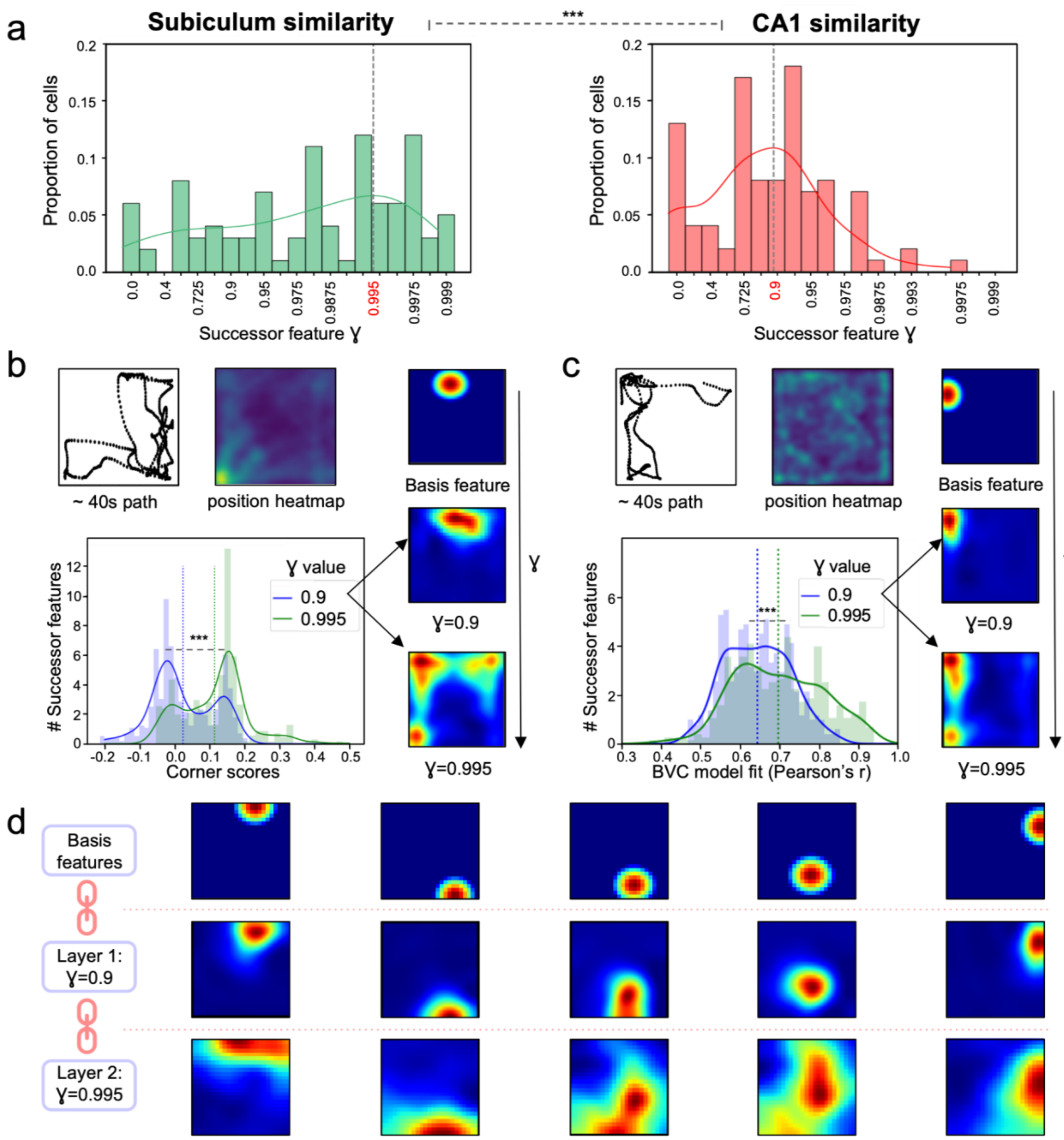

Updated Fig. 7 Distinct temporal horizons capture subicular and hippocampal representations. (**a**) Distribution of best-fit γ values for subicular (left) and CA1 (right) cell responses; kernel density estimate (KDE) shown as solid line, vertical line indicates KDE maximum. Subicular cells were best fit by γ ≈ 0.995 (temporal horizon ~20 s), while CA1 cells were best fit by γ ≈ 0.9 (temporal horizon ~1 s). (**b**) Corner representations emerge with extended temporal horizon. Top: trajectory (~40 s shown) and position heatmap illustrating corner-dwelling behaviour. Right: a corner-proximal basis feature at γ = 0.9 (CA1-like) and γ = 0.995 (subicular-like), demonstrating how extended temporal integration bridges multiple corner locations. Bottom: distribution of corner scores across successor features shifts towards larger values with increasing γ; vertical lines indicate mean values. (**c**) Boundary representations emerge with extended temporal horizon. Top: trajectory (~40 s shown) and heatmap demonstrating thigmotaxic behaviour. Right: a boundary-proximal basis feature transforms into an elongated boundary response at γ = 0.995 (~20 s predictive horizon). Bottom: distribution of boundary vector cell model fits (Pearson's r) shifts towards higher values with increasing γ; vertical lines indicate mean values. *p < 0.05; **p < 0.01; ***p < 0.001. (**d**) Two-stage cascade model in which basis features are first transformed at γ = 0.9 (Layer 1, analogous to CA1) and the resulting successor features then serve as input to a second transformation at γ = 0.995 (Layer 2, analogous to subiculum). Layer 2 produces a substantially greater proportion of corner cells (18% vs 0%) and boundary vector cells (72% vs 7%) than Layer 1, and amplifies both cell types relative to the single-stage model (5–6% corner, 36% BVC at γ = 0.995), indicating that the second stage elaborates predictive structure already present in the first.

Response to Reviewer 3

We are grateful for the reviewer's enthusiasm and positive assessment of our manuscript. Their comment raises an important conceptual question that we are pleased to address.

1. It appears that the authors chose the discount parameter gamma to optimise the similarity between subicular firing fields recorded in experimental data and the firing fields in the simulations. One may ask then, is the choice of this parameter range biologically plausible? Why is the time horizon in subicular representations 16–20 s (and 1–2 s) in CA1 and not some other value? Does the animal not need representations of expected future states at intermediate time horizons? If yes, then where should we look for those? If not, what is special about these two time horizons, or are the exact discount factors or time horizons dependent on the behavioural or environmental context?

This is an excellent and thought-provoking question that we are happy to address.

The CA1 timescale (~1–2s) aligns with theta sequence dynamics: during each ~125ms theta cycle, sequential place cell activity sweeps through several upcoming spatial locations (Dragoi & Buzsáki, 2006). George et al. (2023) demonstrated that theta phase precession combined with spike-timing-dependent plasticity provides a biologically plausible mechanism for learning successor representations, grounding the CA1 timescale in known physiology. Notably, during 2D exploration, within each theta cycle the decoded position sweeps up to approximately one place field width ahead of the animal — in dorsal CA1 this corresponds to roughly 20 cm or less (Muessig et al., 2019), which is approximately the distance a mouse covers in 1–2 s. This spatial horizon aligns well with the fitted $\gamma = 0.9$, suggesting that the effective temporal horizon of the learned representation is set jointly by the reach of theta sweeps and biophysical constraints on the STDP learning window. This is consistent with sensory-timescale prediction frameworks (Whittington et al., 2020).

The longer subicular timescale (~20s) corresponds to the duration of typical behavioural bouts such as thigmotaxic wall-running, consistent with a proposed role for subiculum as encoding predictive information at behaviourally relevant timescales. We acknowledge that we do not yet have a complete account of why these specific absolute timescales emerge. However, the key finding is the relative difference between CA1 and subiculum — subicular representations are consistently best fit by successor representations with longer predictive horizons — which is robust across our analyses and does not depend on the precise absolute values of γ.

Regarding intermediate horizons: we note that the two timescales we report should not be taken as the only predictive horizons present. Our analysis in Fig. 7a shows that while CA1 and subicular recordings are best fit by $\gamma = 0.9$ and $\gamma = 0.995$ respectively overall, individual cells span a range of optimal γ values — consistent with each region expressing a distribution of predictive horizons rather than a single fixed timescale. More broadly, the well-established dorso-ventral gradient in place field size across the hippocampus can be understood as a gradient of predictive timescales (Momennejad & Howard, 2018), with ventral regions encoding longer-horizon predictions than dorsal. Our recordings were made in dorsal hippocampus and subiculum; other combinations of timescales are almost certainly represented at other positions along this axis.

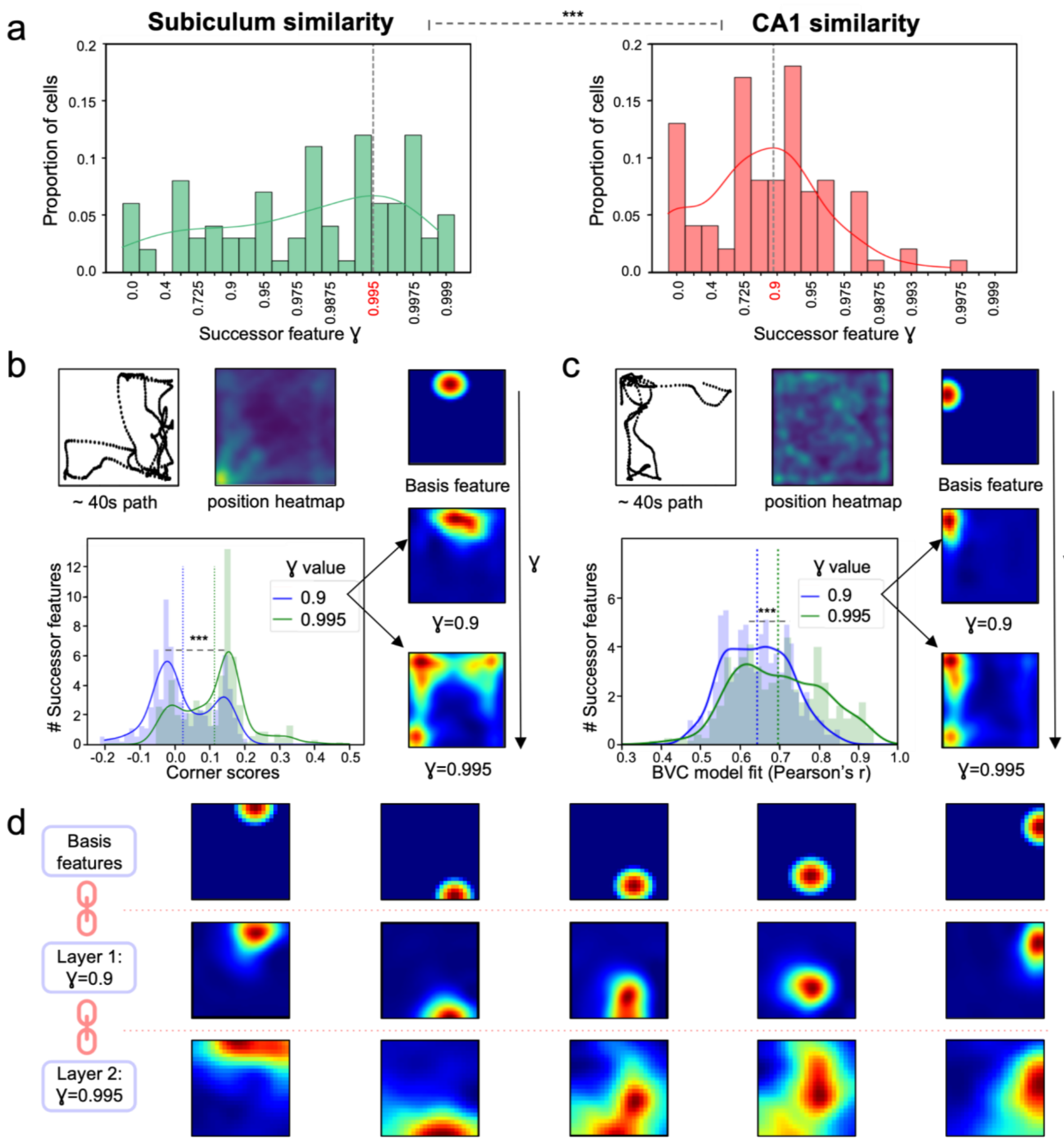

Updated Fig. 7 Distinct temporal horizons capture subicular and hippocampal representations. (**a**) Distribution of best-fit γ values for subicular (left) and CA1 (right) cell responses; kernel density estimate (KDE) shown as solid line, vertical line indicates KDE maximum. Subicular cells were best fit by γ ≈ 0.995 (temporal horizon ~20 s), while CA1 cells were best fit by γ ≈ 0.9 (temporal horizon ~1 s). (**b**) Corner representations emerge with extended temporal horizon. Top: trajectory (~40 s shown) and position heatmap over full 20 minute trial illustrating corner-dwelling behaviour. Right: a corner-proximal basis feature at γ = 0.9 (CA1-like) and γ = 0.995 (subicular-like), demonstrating how extended temporal integration bridges multiple corner locations. Bottom: distribution of corner scores across successor features shifts towards larger values with increasing γ; vertical lines indicate mean values. (**c**) Boundary representations emerge with extended temporal horizon. Top: trajectory (~40 s shown) and heatmap over full 15 minute trial demonstrating thigmotaxic behaviour. Right: a boundary-proximal basis feature transforms into an elongated boundary response at γ = 0.995 (~20 s predictive horizon). Bottom: distribution of boundary vector cell model fits (Pearson's r) shifts towards higher values with increasing γ; vertical lines indicate mean values. *p < 0.05; **p < 0.01; ***p < 0.001. (**d**) Two-stage cascade model in which basis features are first transformed at γ = 0.9 (Layer 1, analogous to CA1) and the resulting successor features then serve as input to a second transformation at γ = 0.995 (Layer 2, analogous to subiculum). Layer 2 produces a substantially greater proportion of corner cells (18% vs 0%) and boundary vector cells (72% vs 7%) than Layer 1, and amplifies both cell types relative to the single-stage model (5–6% corner, 36% BVC at γ = 0.995), indicating that the second stage elaborates predictive structure already present in the first.

We have expanded the Discussion to address these points:

"Our framework extends this, suggesting a multi-level hierarchy where each region computes predictive representations appropriate to its inputs: CA1 builds successor representations over boundary/geometric inputs from entorhinal cortex, while subiculum builds successor representations over the resulting place cell representations. These two levels operate over distinct temporal horizons. The CA1 timescale (~1–2 s) can be grounded in the biophysics of theta phase precession and spike-timing-dependent plasticity[54,78]; during open-field exploration, the decoded position advances up to approximately one place field width ahead of the animal within each ~125 ms theta cycle (~20 cm in dorsal CA1[60]), corresponding to the distance covered in 1–2 s and aligning with the fitted $\gamma = 0.9$. The longer subicular timescale ($\gamma = 0.995$, ~20 s) corresponds to the duration of typical behavioural bouts, consistent with a role for subiculum in encoding the statistical structure of behaviour over extended timescales. This arrangement — short-horizon prediction in CA1, long-horizon prediction in subiculum — mirrors the natural organisation of information flow through the hippocampal-parahippocampal circuit.

These two timescales should not, however, be taken as the only predictive horizons present in the hippocampal formation. Individual cells within both CA1 and subiculum span a range of optimal γ values (Fig. 7a), indicating that each region expresses a distribution of predictive horizons rather than a single fixed timescale. More broadly, the well-established dorso-ventral increase in place field size can be understood as a gradient of predictive timescales[79], with ventral hippocampal regions encoding longer-horizon successor representations than dorsal. Our recordings, drawn from dorsal hippocampus and subiculum, capture two points along what may be a continuous spectrum. Importantly, the central finding — that subicular representations are best approximated by successor representations with longer temporal horizons than those fitting CA1 — is a relative observation that is robust across our analyses and does not depend on the absolute value of γ. Whether the relative ordering of predictive horizons between CA1 and subiculum is maintained under goal-directed or changing behavioural policies — and whether the absolute timescales shift with task demands — represents a key direction for future work. The successor representation was originally formulated to enable rapid revaluation when reward contingencies change, by separating the predictive structure of the environment from current reward values[44]. Our findings demonstrate that this predictive structure alone — independent of reward — is sufficient to account for the heterogeneous spatial responses observed in subiculum. An important open question is how the temporal hierarchy we identify here responds when animals must flexibly adapt their behaviour under changing task demands. In particular, the dynamics of subicular updating under new behavioural policies — and whether the relative ordering of predictive horizons between CA1 and subiculum is preserved — remain to be established experimentally."
